# KRAS^G12R^-Mutant Pancreatic Cancer Features Limited ERK/MAPK Transcriptional Activity and a Distinctive Tumor Microenvironment

**DOI:** 10.1158/0008-5472.CAN-25-2630

**Published:** 2026-01-13

**Authors:** Rachel A. Burge, Ozgun Le Roux, Olesja Popow, Victoria K. Spadafora, Christabelle Rajesh, Sara J. Adair, Lucas Bialousow, Cailey Murphy, Samaneh Saberi, Silvia G. Vaena, Margaret C. Taquey, Sarah Allen, Lu Han, Kristi L. Helke, Sudarshana Sharma, Michael C. Ostrowski, Denis C. Guttridge, Toros A. Dincman, Todd W. Bauer, David F. Kashatus, Thomas McFall, Kevin M. Haigis, Michael A. Hollingsworth, Christopher M. Counter, Channing J. Der, G. Aaron Hobbs

**Affiliations:** 1 Department of Biochemistry & Molecular Biology, Medical University of South Carolina, Charleston, South Carolina.; 2 Department of Pharmacology & Cancer Biology, Duke University Medical Center, Durham, North Carolina.; 3 Department of Cancer Biology, Dana-Farber Cancer Institute, Boston, Massachusetts.; 4 Department of Medicine, Brigham & Women’s Hospital, and Harvard Medical School, Boston, Massachusetts.; 5 Department of Pediatrics, Darby Children’s Research Institute, Medical University of South Carolina, Charleston, South Carolina.; 6 Department of Surgery, University of Virginia, Charlottesville, Virginia.; 7 Bioinformatics Core, Medical University of South Carolina, Charleston, South Carolina.; 8 College of Graduate Studies, Medical University of South Carolina, Charleston, South Carolina.; 9 Hollings Cancer Center, Medical University of South Carolina, Charleston, South Carolina.; 10 Department of Comparative Medicine, Medical University of South Carolina, Charleston, South Carolina.; 11 Division of Hematology and Oncology, Department of Medicine, Medical University of South Carolina, Charleston, South Carolina.; 12 Department Microbiology, Immunology, and Cancer Biology, University of Virginia Health System, Charlottesville, Virginia.; 13 Department of Biochemistry and MCW Cancer Center, Medical College of Wisconsin, Milwaukee, Wisconsin.; 14 Eppley Institute for Research in Cancer and Allied Diseases, Fred and Pamela Buffett Cancer Center, University of Nebraska Medical Center, Omaha, Nebraska.; 15 Lineberger Comprehensive Cancer Center, University of North Carolina at Chapel Hill, Chapel Hill, North Carolina.; 16 Department of Pharmacology, University of North Carolina at Chapel Hill, Chapel Hill, North Carolina.

## Abstract

Patients with pancreatic ductal adenocarcinoma (PDAC) harboring *KRAS*^*G12R*^ mutations have increased overall survival relative to patients with *KRAS*^*G12D/V*^ mutations. To investigate the mechanisms underlying this differential outcome, we developed a genetically engineered mouse model (GEMM) harboring *Kras*^*G12R*^ and Tr*p53*^*R172H*^ mutations (*Kras*^*LSL-G12R/+*^*;Trp53*^*LSL-R172H/+*^*;p48*^*Cre-ERTM*^). Unlike *Kras*^*G12D*^ models, *Kras*^*G12R*^ GEMMs exhibited limited tumorigenesis, with only 10% developing pancreatic tumors after 1 year. Additionally, mice harboring whole-body expression of *Kras*^*G12R*^ remained healthy for over 1 year, whereas *Kras*^*G12D*^ mice developed rapid multifocal disease. Comparison of KRAS mutant-selective transcription and signaling in murine and human PDAC cell lines, GEMMs, and patient-derived xenograft (PDX) mouse models revealed that direct KRAS-mediated PI3K activation is necessary for robust tumor initiation in GEMMs. Unexpectedly, KRAS was not the primary driver of PI3K activity in human PDAC cell lines and PDX models, regardless of KRAS mutation. KRAS^G12R^ and KRAS^G12D^ activated a similar pancreas-specific transcriptional network, but KRAS^G12R^ promoted these pathways less robustly due to limited ERK/MAPK nuclear translocation. Finally, *KRAS*^*G12R*^ human pancreatic tumors had an altered tumor microenvironment (TME) with reduced collagen deposition and metastatic liver invasion. Together, this study demonstrated that KRAS^G12R^ is capable of driving tumorigenesis despite the reduced ERK/MAPK nuclear translocation and transcriptional output. Although human KRAS^G12D^- and KRAS^G12R^-mutant tumors display unexpected similarities in PI3K activity, the differential ERK/MAPK signaling activity and the extrinsic consequences on the TME provide support for using *KRAS*^*G12R*^ mutation status as a prognostic biomarker for therapeutic strategies.

**Significance:**

KRAS^G12R^-mutant pancreatic cancer is characterized by lower ERK/MAPK nuclear translocation and transcriptional output than KRAS^G12D^-mutant tumors, offering a potential window for patients with KRAS^G12R^ mutations to derive additional benefit from neoadjuvant therapy.

*See related commentary by Tiriac and Engle, p. 1817*

*See related article by Burge et al., p. 1854*

*See related article by Kamgar et al., p. 2042*

## Graphical Abstract

**Figure:**
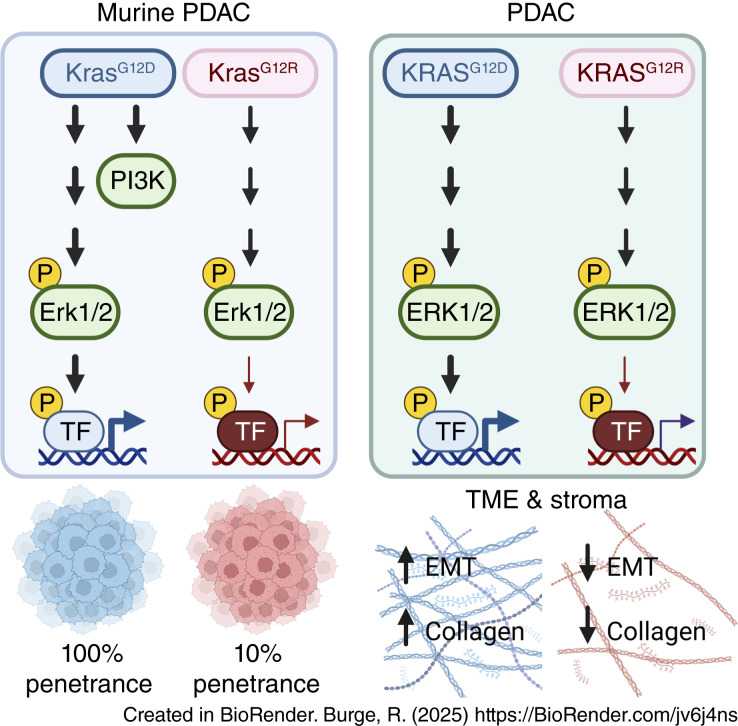

## Introduction

Pancreatic cancer has the lowest 5-year survival rate among major organ cancers (13%) and ranks as the third leading cause of cancer mortality in the United States (1). Pancreatic ductal adenocarcinoma (PDAC) comprises 90% of all pancreatic cancer cases (2). Despite a well-defined genetic landscape, no clinically effective targeted therapies have been identified for PDAC, with the current first-line standard-of-care chemotherapy comprising conventional cytotoxic drug combinations (3).

*KRAS* mutations are considered the initiating event for PDAC development and occur in approximately 95% of PDAC cases (4, 5). *KRAS* gain-of-function hotspot point mutations occur at residues G12, G13, and Q61. The most frequent *KRAS* missense mutations in PDAC are *KRAS*^*G12D*^ (42%), *KRAS*^*G12V*^ (31%), and *KRAS*^*G12R*^ (15%; GENIE Cohort version 17.0). By comparison, the frequency and spectrum of these specific *KRAS* mutations in pancreatic intraepithelial neoplasia (PanIN) lesions are comparable with those in PDAC, suggesting that selection for *KRAS* mutation occurs at PanIN initiation rather than during tumor progression (4, 6).

The biochemical and molecular basis of tissue-distinct *KRAS* mutational patterns in cancer remains poorly understood. The prevalence of *KRAS*^*G12C*^ mutations in lung and colorectal cancer can be attributed almost exclusively to current or former smokers (7). However, two studies compared projected *KRAS* mutational frequencies based on observed DNA mutation frequencies versus actual frequencies found in patients with cancer and concluded that there is a strong biological basis for the occurrence of specific *KRAS* mutations across a range of *RAS*-mutant cancers (8, 9). In contrast to their prevalence in PDAC, *KRAS*^*G12R*^ mutations are found in only 1% to 2% of non–small cell lung and colorectal cancers.

There is increasing evidence that different point mutations have distinct functional consequences on KRAS function as a cancer driver; consequently, distinct therapeutic approaches may be more effective for distinct KRAS-mutant cancers (10). Among the most unique mutations is *KRAS*^*G12R*^, a prime example of mutant-selective differential downstream effector signaling and is specifically defective in binding to the catalytic subunit of the lipid kinase PI3Kα (p110a; encoded by *PIK3CA*; ref. 11). Unlike other KRAS mutants, we determined that KRAS^G12R^ is unable to drive wild-type (WT) RAS activation via the RAS guanine exchange factor SOS1 allosteric activation site (11). Unlike KRAS^G12D/V^, KRAS^G12R^ did not inhibit the RasGAP NF1 (12). As genetically engineered mouse models (GEMM) have demonstrated that mutant KRAS interaction with PI3Kα is necessary for lung cancer initiation and maintenance (13, 14), and ablation of *Pik3ca* in the pancreas results in reduced tumorigenesis (15), it is surprising that the *KRAS*^*G12R*^ mutation is prevalent in human PDAC. Finally, unlike all other G12 mutations evaluated, KRAS^G12R^ is insensitive to recently developed GDP-selective pan-KRAS inhibitors (16).

Evaluation of patients with PDAC supports mutation-specific differences in *KRAS* oncogenic potential (5, 17). Patients with *KRAS*^*G12R*^-mutant PDAC were more likely to be early-stage (stage I) compared with *KRAS*^*G12D*^ (44% vs. 24%; ref. 17). *KRAS*^*G12R*^-mutant PDAC also showed better overall survival than *KRAS*^*G12D*^-mutant PDAC at 39 months versus 30 months. Finally, in patients who were candidates for surgical resection, *KRAS*^*G12R*^-mutant PDAC was more often lymph node-negative than *KRAS*^*G12D*^-mutant PDAC (47% and 26%, respectively).

Despite its frequency in human PDAC, *Kras*^*G12R*^ has so far been unable to initiate PDAC in autochthonous murine models (18). Therefore, we generated multiple *Kras*^*G12R*^-mutant GEMMs to better understand the limitations of the Kras^G12R^ mutant. *Kras*^*G12R*^ GEMMs showed resistance to tumor initiation, with tumors detected in only 10% of mice between 12 and 18 months of age. However, these models demonstrate for the first time that *Kras*^*G12R*^ is capable of driving tumorigenesis in GEMMs. We then demonstrate that Kras^G12D^ directly promotes PI3K signaling in murine models, providing rationale for the relative weakness of Kras^G12R^ in murine models. Using RNA sequencing (RNA-seq) data from cell model systems and patient-derived xenografts (PDX), we determined that Kras^G12R^ is also significantly weaker at driving hallmark KRAS transcriptional signatures and ERK nuclear translocation in comparison with KRAS^G12D^. Further, we demonstrate that *KRAS*^*G12R*^-mutant cell lines have significantly decreased migration capacity, and collagen deposition in *KRAS*^*G12R*^ tumors from human patients is significantly reduced compared with *KRAS*^*G12D*^ tumors. Collectively, these findings reinforce that the canonical model of KRAS-mediated signaling is only required in murine PDAC and demonstrate that direct KRAS-PI3K signaling is not required in human PDAC, regardless of KRAS mutation. These data highlight that the human pancreas offers a uniquely susceptible environment for *KRAS*^*G12R*^-mediated tumorigenesis, and the reduced ERK/MAPK signaling, slower metastasis, and altered collagen profile provide a window for which neoadjuvant therapy would offer an increased benefit to patients with* KRAS*^*G12R*^ PDAC.

## Materials and Methods

### Generation of mouse models

All mouse procedures were approved by the Institutional Animal Care and Use Committees of Duke University (A195-19-09), MUSC (2020-01071), and BIDMC (081-2017). We engineered a *Kras*^*LSL-G12R/+*^ allele and generated models using mice from The Jackson Laboratory (see Supplementary Data). Cre-mediated recombination was induced by tamoxifen injection at specified time points. Mice were housed under standard conditions with *ad libitum* access to chow and water. Euthanasia and endpoint criteria followed approved protocols. Both sexes were used in roughly equal ratios. For mouse embryonic fibroblasts (MEF), one male and one female mouse were housed in the same cage, and the development of plugs was monitored daily. Embryos were harvested as previously described (19). More extensive details can be found in the Supplementary Data and in Supplementary Table S1.

### Ras activity assays in MEF cultures

Two independent cell lines from *Kras*^*LSL-G12R/+*^ and WT MEFs stably expressing Cre were used for GTP pull-down and signaling assays using the Active Ras Detection Kit (Cell Signaling Technology, #8821) according to the manufacturer’s protocol.

### Cell lines and cell culture conditions

The VMP-654, VMP-366, VMP-188, and VMP-395T cell lines were generously provided by D.F. Kashatus and T.W. Bauer (University of Virginia), as previously described (20, 21). KP-2 (RRID: CVCL_3004) and TCC-PAN2 (RRID: CVCL_3178) were obtained from the Japanese Collection of Research Bioresources Cell Bank. PK-8 (RRID: CVCL_4718) cells were obtained from the RIKEN Cell Bank. Pa16C cells were provided by A. Miatra (MD Anderson) as described previously (22). Primary MEFs expressing only KRAS (DU1473; Hras^−/−^;Nras^−/−^;Kras^lox/lox^;RERT^ert/ert^) were generated and characterized previously (23) and were provided by the NCI RAS Initiative. The remaining cell lines were obtained from the ATCC: AsPC-1 (RRID: CVCL_0152), HPAC (RRID: CVCL_3517), HPAF-II (RRID: CVCL_0313), PANC1 (RRID: CVCL_0480), SW1990 (RRID: CVCL_1723), PSN1 (RRID: CVCL_1644), and HuP-T3 (RRID: CVCL_1299). Conditions for each cell line are available in the supplemental files (Supplementary Table S2). All cell lines were maintained in either high glucose DMEM or RPMI 1640 supplemented with 10% FBS. All cell lines were maintained in a humidified chamber with 5% CO_2_ at 37°C. Unless otherwise indicated, cells were serum-starved overnight prior to immunoblot analysis. Cell lines used in experiments were passaged for approximately 10 to 15 passages before a new aliquot was thawed. Cell line authenticity was verified by short tandem repeat profiling for cell lines for which short tandem repeat data are available, and all lines were monitored monthly for *Mycoplasma* contamination using the Lonza MycoAlert Mycoplasma Detection Kit (cat. #LT07-218).

### Human pancreatic cancer samples

Human pancreatic cancer samples for PDX experiments were obtained in accordance with the University of Virginia Institutional Review Board (IRB) for Health Sciences Research under the direction of T.W. Bauer. Collection of human PDAC specimens was performed with the approval of the IRB at the University of Virginia in coordination with the Biorepository and Tissue Research Facility (University of Virginia, IRB-HSR 13529). All patients provided written informed consent for participation and conducted in accordance with the U.S. Common Rule. No patient received neoadjuvant therapy. This study was carried out in strict accordance with the recommendations in the Guide for the Care and Use of Laboratory Animals of the NIH. The protocol was approved by the Animal Care and Use Committee of the University of Virginia (PHS Assurance, #A3245-01). Primary PDX tumors growing in the pancreas were harvested as previously described (21, 24). Human pancreatic cancer samples from the Hollings Cancer Center were obtained under the direction of T.A. Dincman. All patients provided written informed consent for participation, conducted in accordance with the U.S. Common Rule. KRAS mutation status was determined using Sanger sequencing (Eurofins Genomics) or next-generation sequencing.

### Immunoblotting

Immunoblotting was performed using a standard protocol described previously (11). To determine the levels of activated proteins, blot analyses were performed using the antibodies listed in Supplementary Table S2.

### Tissue analyses

Mice were euthanized and underwent full necropsy. Selected organs and tumors were fixed in 10% formalin, processed, embedded in paraffin, sectioned at 5 μm, and stained with hematoxylin and eosin (H&E) or immunofluorescence. Routine processing was performed as described in Supplementary Methods. IHC and special stains [Masson’s Trichrome, Picrosirius red (PSR)] followed standard protocols. Slides were scanned and analyzed using Akoya Biosciences software and ImageJ. Further details, including antibody information and full protocols, are available in the Supplementary Data.

### RNA-seq

Sequencing libraries for the human pancreatic nestin-expressing (HPNE) cell lines were prepared at the Medical University of South Carolina Translational Science Lab as described in the Supplementary Data. PDX model RNA-seq was conducted at the University of Virginia as described in the Supplementary Data and is available from GSE252909. Gene set enrichment analysis (GSEA) was performed using the GSEA desktop application (version 4.3.3; Broad Institute; https://www.gsea-msigdb.org). Differentially expressed genes were ranked by expression changes between conditions, and normalized enrichment scores (NES) and false discovery rates (FDR, *q* values) were calculated as described (25).

### Scratch-wound migration assay

The cell lines were seeded in six-well plates and allowed to proliferate until they reached 100% confluence. The confluent cells were wounded by scraping with a sterile 200 μL pipette tip both horizontally and vertically. Data were normalized to the 0-hour time point, and statistical analysis was performed using a Student *t* test to compare differences in wound closure rates between cell lines. All analyses were performed using ImageJ software.

### Bioluminescence resonance energy transfer assays

HEK293T cells were seeded in 96-well white opaque plates and cotransfected with CRAF-NanoLuc and varying amounts of GFP-tagged KRAS mutants using Lipofectamine 3000. After 24 hours, Nano-Glo Live Cell Reagent was added, and luminescence was measured using a Tecan Infinite M200 PRO. Bioluminescence resonance energy transfer (BRET) ratios were calculated from dual emission readings and plotted against the RAS-GFP/NF1-NanoLuc ratio. Assays were performed in triplicate with eight biological replicates each. Additional details are provided in the Supplementary Methods.

### Statistical analysis

Data were analyzed using the GraphPad Prism built-in test (one-way ANOVA, Dunnett multiple comparisons test) or Student *t* test in R 4.2.2. Data are presented relative to the respective controls or as noted in the figure legend. Error bars indicate mean ± SEM for ≥3 independent experiments (except where noted), and *P* values on graphs are denoted by *, *P* < 0.05; **, *P* < 0.002; ***, *P* < 0.0005; and ****, *P* < 0.0001 or as indicated in each figure legend. The number of samples analyzed per experiment and whether the data are representative or averaged are indicated in the figure legend.

## Results

### Postnatal Kras^G12D^, but not Kras^G12R^, activation drives invasive and metastatic murine PDAC

**Figure 1.**
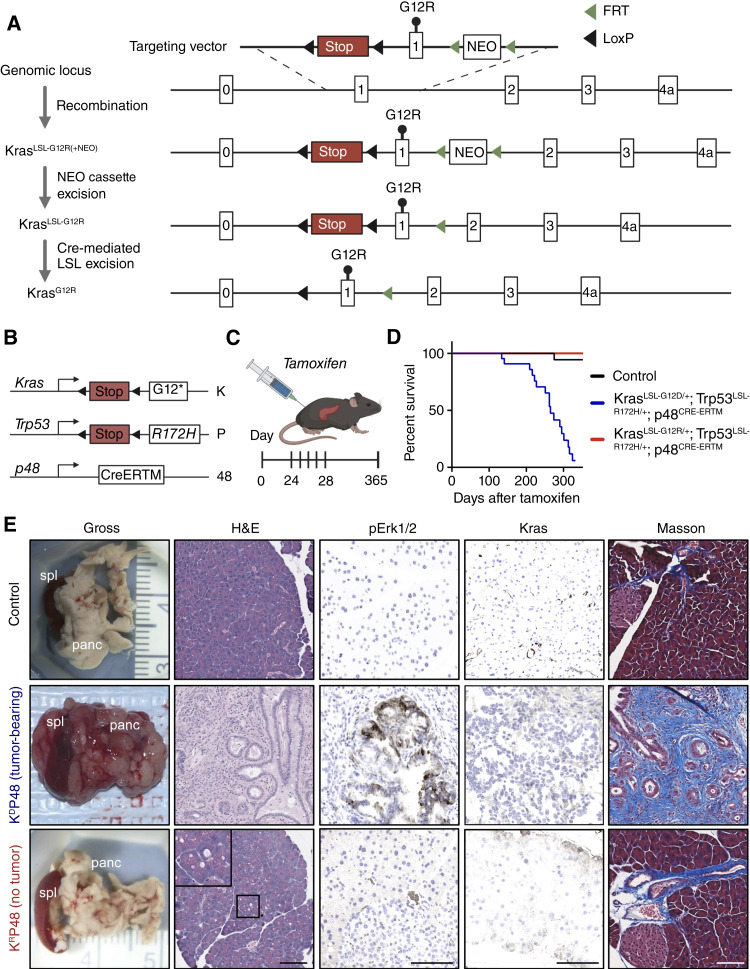
Pancreas-specific coexpression of *Kras*^*LSL-G12D/+*^*;Trp53*^*LSL-R172H/+*^, but not *Kras*^*LSL-G12R/+*^*;Trp53*^*LSL-R172H/+*^, drives invasive PDAC. **A,** Schematic of the generation of the Kras^LSL-G12R^ allele. NEO, neomycin resistance cassette and was used as a selectable marker. **B,** Schematic of the *Kras*^*LSL-G12R/+*^*;Trp53*^*LSL-R172H/+*^*;p48*^*Cre-ERTM*^ mouse model (KP48). **C,** Mice and littermate controls (24–28 days old) received tamoxifen (1 mg/10 g body weight) via i.p. injection for five consecutive days. **D,** Kaplan–Meier survival curves for controls (*n* = 26), K^D^P48 (*n* = 26), and K^R^P48 (*n* = 20) mice. **E,** Gross images of the pancreas (panc) and spleen (spl). Pancreas sections were additionally stained with H&E, immunostained for pErk1/2 and Kras, and stained with Masson’s trichrome. Images are representative of six mice from each cohort (three male; three female). Immunostained markers were false-colored brown for visualization. H&E images were analyzed by a veterinary pathologist. Scale bar, 100 μm. Created in BioRender. Burge, R. (2025) https://BioRender.com/jv6j4ns.

*KRAS* ^G12R^ is the third most prevalent KRAS mutation in PDAC (15%; GENIE version 17.0). Therefore, we generated a Cre-inducible *Kras*^*LSL-G12R*^ allele (Fig. 1A). To validate the expression and activity of *Kras*^*LSL-G12R*^, we derived two MEF cell lines and validated the recombination of the *Kras*^*LSL-G12R*^ allele (Supplementary Fig. S1A and S1B). Using the RAF1 RAS-binding domain pull-down assay, we showed an increase in the GTP-loaded Kras fraction after mutant Kras^G12R^ recombination (Supplementary Fig. S1C–S1E). We confirmed that our *p48*^*Cre-ERTM*^ promoted Cre-mediated recombination in the pancreas using two *Kras*^*LSL-G12R/+*^*;p48*^*Cre-ERTM*^*;Rosa26*^*Tomato*^ mice (Supplementary Fig. S1F and S1G).

As previous *Kras*^*LSL-G12R/+*^*;p48-Cre* GEMMs failed to develop PDAC after 1 year (18), we speculated that the later-stage activation of the Kras allele under the control of *p48*^*Cre-ERTM*^ would better represent the onset of human PDAC development. We generated *Kras*^*LSL-G12R/+*^*;Trp53*^*LSL-R172H/+*^*;p48*^*Cre-ERTM*^ (designated K^R^P48) mice alongside *Kras*^*LSL-G12D/+*^*;Trp53*^*LSL-R172H/+*^*;p48*^*Cre-ERTM*^ (K^D^P48) mice as a positive control (Fig. 1B and C). Our KP48 model is the first *Kras;Trp53*-mutant GEMM to utilize a *p48*^*Cre-ERTM*^ promoter. The majority of the K^D^P48 GEMMs exhibited severe physiologic distress by 200 days, with an average survival time of ∼250 days. In contrast, K^R^P48 mice showed no obvious signs of distress at 1 year of age (Fig. 1D). The healthy appearance of the K^R^P48 mice was unexpected, given that the orthotopic *Kras*^*G12R*^*;Trp53*^*fl/fl*^ model resulted in significant tumor burden (18).

H&E staining of K^D^P48 pancreas tissue identified histologic features characteristic of PDAC, with increased collagen deposition and pErk activation. The majority of the K^R^P48 mice showed no PanIN lesions, only an increased presence of vacuoles and fibrosis around ducts in the pancreas (K^R^P48 no tumor; Fig. 1E). We conclude that the majority of K^R^P48 mice are resistant to tumorigenesis even at an advanced age of onset, but it remains undetermined whether *Kras*^*G12R*^ is driving senescence or is too weak to induce tumorigenesis.

### Kras^G12R^ induces hyperplasia and vacuole formation in the pancreas

**Figure 2.**
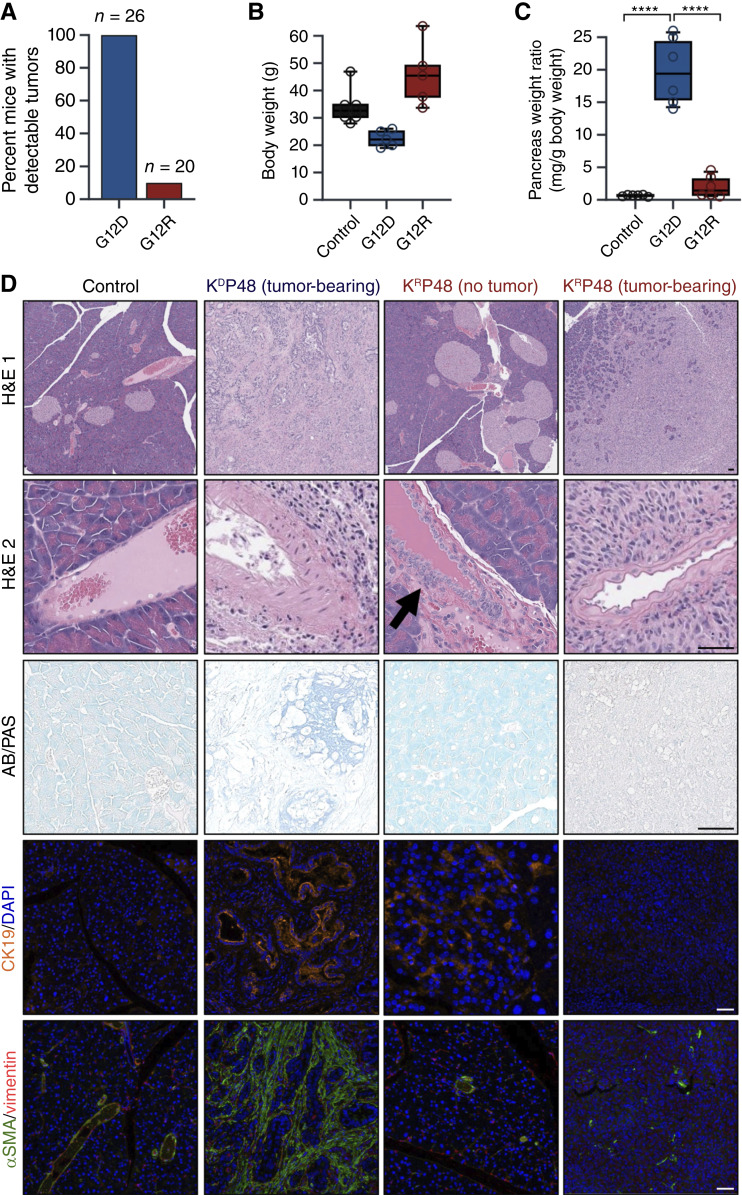
*Kras* ^*LSL-G12R/+*^ induced pancreatic hyperplasia and vacuole formation with limited tumor development after 1 year. **A,** Graph showing the percentage of KP48 mice that developed pancreatic tumors. The number of mice (*n*) is indicated above each corresponding bar. **B,** Box plot of terminal body weights of male mice that were sacrificed at the determined endpoint and had tissues harvested. K^D^P48 mice (*n* = 6), K^R^P48 mice (*n* = 6), and control mice (*n* = 6). Female mice followed similar trends. Data are presented as mean weight at endpoint with no statistically significant differences in body weight. **C,** Box plot of pancreas weights normalized to total mouse weight at the time of sacrifice. K^D^P48 mice had significantly larger pancreata in comparison with control and K^R^P48 mice. *P* values were determined using one-way ANOVA with Tukey multiple comparison test. ****, *P* < 0.0001. **D,** Representative gross images of the pancreas. K^R^P48 (no tumor) has an increase in islet cells (H&E 1) and proliferation of ductal epithelial cells (H&E 2, black arrow). Both K^D^P48 (tumor-bearing) and K^R^P48 (tumor-bearing) mice show increased periductal fibrosis. Representative immunostains for CK19 (ductal marker), αSMA (fibroblast), Ki-67 (proliferation), and Alcian blue–periodic acid-Schiff (AB-PAS) stain (glycogen and zymogen granules). H&E images were analyzed by a veterinary pathologist. Scale bar, 50 μm.

Although 100% of K^D^P48 mice developed metastatic PDAC, extensive histologic analysis at the study endpoint revealed that only 10% of K^R^P48 mice formed pancreatic tumors (K^R^P48 tumor-bearing; Fig. 2A). The only two mice that formed tumors were collected at the endpoint of this study, by which time the tumors had progressed beyond the PanIN stage. All other K^R^P48 mice had no detectable PanIN or other lesions. At the tumor burden endpoint, the body weight of K^D^P48 mice trended lower compared with controls (Fig. 2B), and their pancreata were significantly larger than those of K^R^P48 and control mice (Fig. 2C). Consistent with previous findings utilizing similar murine models (26), K^D^P48 mice developed locally invasive PDAC, with elevated expression of CK19 (ductal marker), αSMA, and vimentin (fibroblast markers; Fig. 2D). The nontumor-bearing K^R^P48 mice exhibited a heterogeneous phenotype marked by enlarged and increased islets, exocrine vacuolation, and ductal hyperplasia, as seen by H&E staining (Fig. 2D). The islet cell expansion reflects a Kras^G12R^-associated phenotype, as it was also evident in the *Kras*^*LSL-G12R/+*^*;p48*^*Cre-ERTM*^ (K^R^48) pancreas (Supplementary Fig. S2A). H&E staining of tumors from the two K^R^P48 mice revealed a distinct, highly cellular morphology with minimal fibrous stroma. Consistent with this observation, *Kras*^*G12R*^ tumor-bearing mice exhibited reduced expression of fibroblast markers compared with *Kras*^*G12D*^ tumor-bearing controls. Alcian blue–periodic acid-Schiff staining, which labels acid mucins blue and neutral mucins pink, revealed distinct mucin profiles between tumors from G12D- and G12R-bearing mice (Fig. 2D).

K^D^P48 mice exhibited increased stromal infiltration in the liver, consistent with metastatic spread and hepatic necrosis (Supplementary Fig. S2B). In the K^R^P48 mice, we observed cytoplasmic rarefaction in the liver, suggestive of excessive glycogen accumulation or steatosis (27). Because both experimental and aged control mice were hemizygous for Trp53 expression and displayed similar pathology, we conclude that this phenotype likely results from the loss of one *Trp53 *allele. In agreement, rarefaction was not observed in an age-matched K^D^P48 animal with both copies of Trp53 intact (Supplementary Fig. S2C).

Although mutant *KRAS*-dependent PDAC is dependent on hyperactivation of ERK signaling (28), excessive ERK signaling promotes senescence and apoptosis (29). Biochemically, KRAS^G12R^ has reduced GTP dissociation and hydrolysis rates, leading to higher levels of GTP loading compared with other Gly12 mutants (30). Thus, we questioned whether oncogenic fitness or initiation of Kras^G12R^ GEMMs was limited due to excessive Mek/Erk MAPK pathway activation. We compared one of the tumor-free K^R^P48 mice with one that developed an exocrine tumor, in which Mek/Erk signaling was elevated in the tumor-bearing mouse (Supplementary Fig. S2D). However, in *Kras*^LSL-G12^*^/+^*;p48*^*Cre-ERTM*^ mice, comparable staining of activated Mek was observed three weeks after tamoxifen induction (Supplementary Fig. S2E). Thus, overactivation of Mek/Erk is unlikely to be the basis for the inability of *Kras*^*G12R*^ to drive PDAC formation in the murine PDAC model.

### Postnatal ubiquitous KRAS^G12D^ but not KRAS^G12R^ expression induces widespread tissue pathology and rapid morbidity

**Figure 3.**
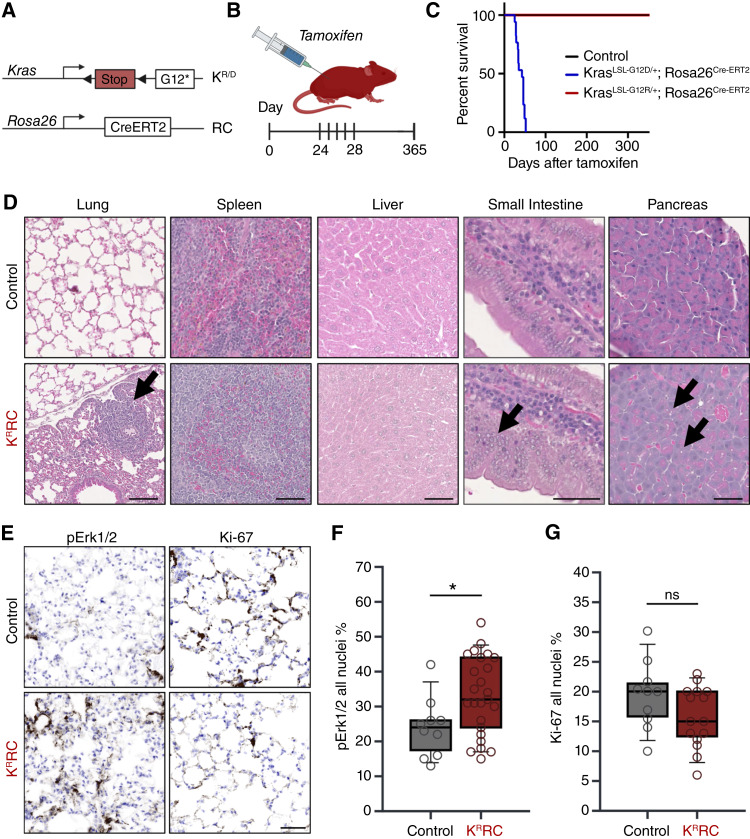
Whole-body *Kras*^*LSL-G12R*^ expression via *Rosa26*^*Cre-ERT2*^ drives heterogeneous hyperplasia in multiple tissues and increases endocrine pancreatic vacuole formation. **A,** Schematic of the K^R^RC. **B,** K^R^RC mice and littermate controls between 24 and 28 days received tamoxifen at 1 mg/10 g of body weight for 5 consecutive days via i.p. injections. **C,** Kaplan–Meier survival curves for K^D^RC mice: *n* = 11 (seven males and four females), *Rosa26*^*Cre-ER/LSL-EYFP*^ control: *n* = 6 (three males and three females), K^R^RC mice: *n* = 5 (two males and three females), and K^R^RC controls: *n* = 2 (one male and one female). **D,** H&E staining of K^R^RC mouse tissues from the lung, spleen, liver, small intestine, and pancreas. A small tumor was detected in a single lobe of the lung in one K^R^RC mouse (black arrow). No major differences were observed in the spleen and liver between K^R^RC and control mice. Hyperplasia of the small intestine was observed in 2/5 K^R^RC mice (black arrow). K^R^RC mice also exhibited increased vacuole presence within exocrine cells of the pancreas (black arrow). Scale bar, 50 μm. **E,** Lung sections were immunostained for pErk1/2 and Ki-67 and false-colored. Images are representative of two Rosa26^Cre-ERT2^ control mice (one male and one female) and five K^R^RC mice (three males and two females). Scale bar, 100 μm. **F,** Box plot showing the average intensity of pErk1/2 in *Rosa26*^*CreERT2/+*^ and *Kras*^*LSL-G12R/+*^*;Rosa26*^*Cre-ERT2/+*^ mice. Five fields of view were taken from each lung. *P* values were calculated using a Student *t* test comparing control mice with G12R. *, *P* < 0.05. Error bars, mean ± SEM. **G,** Box plot showing the average intensity of Ki-67 in the pancreas of *Rosa26*^*Cre-ERT2/+*^ and *Kras*^*LSL-G12R/+*^*;Rosa26*^*Cre-ERT2/+*^ mice. Five FOV from each mouse (two control and three K^R^RC). *P* values were calculated using a Student *t* test comparing control mice with K^R^RC. Error bars, mean ± SEM. All statistical tests were run using R 4.2.2. ns, nonsignificant. Created in BioRender. Burge, R. (2025) https://BioRender.com/jv6j4ns.

Previously, KRAS^Q61R^, which is considered to be a stronger oncogene due to a further depressed GTP hydrolysis rate, was demonstrated to be highly tumorigenic in numerous tissues using the *Rosa26*^*CreERT2/+*^ murine model (19). Therefore, we crossed the *Kras*^*LSL-G12R/+*^ allele into a *Rosa26*^*Cre-ERT2/+*^ background (denoted as K^R^RC), which expresses tamoxifen-inducible Cre in a broad spectrum of tissues (31). Beginning at P24, mice and littermate controls were treated with tamoxifen by five consecutive daily i.p. injections at 1 mg tamoxifen/10 g body weight (Fig. 3A and B). All five K^R^RC mice survived to 1 year of age with no evidence of outward abnormalities (Fig. 3C). As a positive control, we generated a *Kras*^*LSL-G12D/+*^;*Rosa26*^*Cre-ERT2/LSL-EYFP*^ (K^D^RC) model, which received a single i.p. tamoxifen injection at P56 in an attempt to prolong K^D^RC survival and enable comparisons without age-related confounding factors. K^D^RC mice had an average survival time of ∼35 days after tamoxifen injection and exhibited widespread histopathologic abnormalities, including salivary tumors and splenomegaly. H&E staining of the K^D^RC mice confirmed irregular spleen architecture, expanded liver sinusoids, inflammation in the pancreas, and hyperplastic lung lesions (Supplementary Fig. S3A).

Of the five K^R^RC mice, one male developed a small lung tumor, one female displayed perivascular pigment-laden macrophages, and two male mice had areas of hyperplasia in the small intestine. Notably, the pancreas of these mice had an increased presence of vacuoles within exocrine cells and exocrine granules (Fig. 3D). Two mice showed fibrosis around their pancreatic ducts.

All K^D^RC mice had detectable levels of hyperplasia in the lungs, in contrast to only one K^R^RC mouse. Interestingly, the lungs of the K^R^RC mice showed a significant increase in activated Erk1/2 but not in proliferation, as monitored by the proliferation marker Ki-67 (Fig. 3E–G). Thus, despite clear activation of Erk signaling, *Kras*^*G12R*^ was unable to promote consistent tumorigenesis in the K^R^RC *de novo* murine model in mice aged up to 1 year.

In summary, by providing an extended duration for the Kras^G12R^ GEMMs to express mutant Kras, we were able to detect *de novo* tumors driven by the Kras^G12R^ allele in mouse models for the first time. We conclude, based on the extended survival and reduced incidence of hyperplasia and tumor formation in multiple tissues at 1 year of age, that *Kras*^*G12R*^ is reduced in its capacity to initiate tumorigenesis in GEMMs. Additionally, the pancreatic tumors observed in the K^R^P48 mice (2 out of 20) did not resemble typical PDAC morphology.

### Murine models rely on KRAS for PI3K activation and signaling

We next wanted to compare the histology of our GEMMs with human PDAC. We examined the pancreas from KP48 mice and human KRAS^G12D/R^-mutant PDAC via H&E. The tumors from the K^R^P48 GEMM exhibited a morphology distinct from both K^D^P48 GEMMs and human PDAC (Supplementary Fig. S3B). As KRAS^G12D^ PDX models display histologic architecture and stromal composition similar to their human counterparts (21), we questioned whether KRAS^G12R^ PDX models might differ as well. However, broad histologic assessment revealed that KRAS^G12R^ PDX tumors closely resembled tumors from both Kras^G12D^ GEMMs and KRAS^G12D^ human PDAC (Supplementary Fig. S3C). Tumors from the K^R^P48 model did not replicate the histopathology of human PDAC or PDX-derived orthotopic models (Supplementary Fig. S3B and S3C). This, in combination with the reduced tumorigenic potential of *Kras*^*G12R*^ observed in murine initiation models, suggests that Kras-mutant GEMMs may not accurately reflect human PDAC tumors.

**Figure 4.**
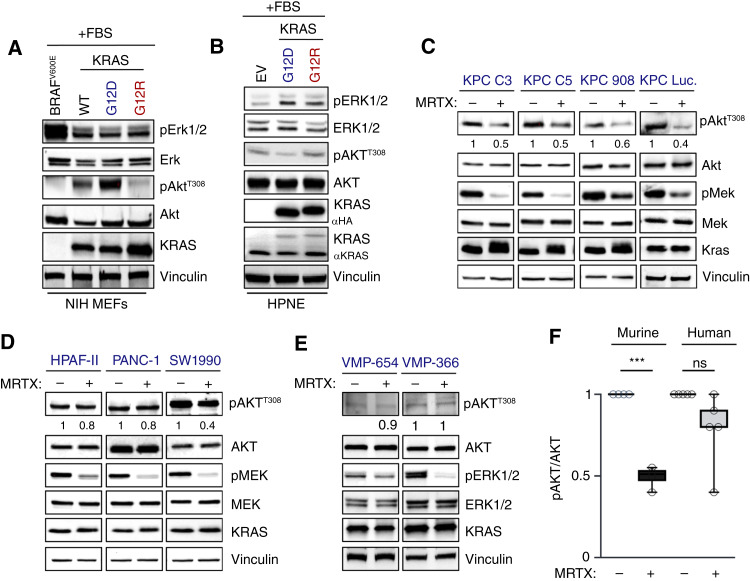
KPC cell lines utilize direct KRAS-PI3K–mediated signaling. **A,** Immunoblot of KRAS mutants expressed in hTERT-HPNE E6/E7 cell lines for 48 hours with doxycycline-induced KRAS expression. The image is representative of three independent experiments. **B,** Immunoblot of NIH “RASless” MEFs expressing BRAF^V600E^ and KRAS^WT/G12D/R^. The image is representative of three independent experiments. **C,** Immunoblot analysis from murine KPC cell lines after inhibition using the KRAS^G12D^-specific inhibitor MRTX1133 at 20 nmol/L for 4 hours. Quantification of pAKT^T308^ after inhibition is shown underneath the blot. Blots are representative of two independent experiments. **D,** Immunoblot analysis of human *KRAS*^*G12D*^ PDAC cell lines after inhibition with the KRAS^G12D^-specific inhibitor MRTX1133 at 20 nmol/L for 4 hours. Quantification of pAKT^T308^ after inhibition is shown underneath the blot. Data are representative of three independent experiments. **E,** Immunoblots with human *KRAS*^*G12D*^ PDX VMP PDAC cell lines after inhibition with the KRAS^G12D^-specific inhibitor MRTX1133 at 20 nmol/L for 4 hours. Quantification of pAKT^T308^ after inhibition is shown underneath the blot. Data are representative of two independent experiments. **F,** Box-and-whisker plot with quantification of pAKT^T308^ in murine PDAC from **C** and human PDAC from **D** and **E**. *P* values were calculated using a Student *t* test comparing MRTX1133-treated cells with vehicle-treated cells. ***, *P* < 0.0002; ns, nonsignificant, *P* > 0.05. Error bars, mean ± SEM. Statistical tests were run using R 4.2.2.

To explore KRAS signaling across murine and human systems, we compared the proliferation of “RASless” MEFs and HPNE cells expressing KRAS^G12D^ or KRAS^G12R^. In both models, KRAS^G12R^-expressing cells proliferated significantly more slowly than their KRAS^G12D^-mutant counterparts (Supplementary Fig. S4A–S4C). We then examined the RAS-MEK-ERK and RAS-PI3K-AKT pathways via immunoblot in full-serum conditions. Expectedly, pERK levels were similar in KRAS^G12D^- and KRAS^G12R^-mutant MEFs and HPNE cells (Fig. 4A and B; Supplementary Fig. S4D and S4E). However, KRAS^G12D^-mutant “RASless” MEFs, but not KRAS^G12D^-mutant HPNE cells, exhibited significantly increased pAKT levels (Fig. 4A and B; Supplementary Fig. S4F). In agreement, RAC1-GTP pull-downs in KRAS^G12R^ “Rasless” MEFs showed a decrease in RAC1-GTP loading, which was not observed in the HPNE KRAS^G12R^ cells (Supplementary Fig. S4G and S4H).

We then utilized the KRAS^G12D^-selective inhibitor MRTX1133 to investigate KRAS-mediated PI3K signaling in murine and human pancreas cancer cell lines (CCL; Fig. 4C–E). KRAS^G12D^ inhibition only reduced pAKT in murine-derived *Kras*^*LSL-G12D/+*^; *Trp53*^*LSL-R172H/+*^*; Pdx1-Cre* (KPC) cell lines but not in most human PDAC cell lines (Fig. 4F). Only the SW1990 cell line showed a consistent decrease in pAKT levels upon KRAS^G12D^ inhibition. Thus, we conclude that PI3K signaling is more dependent on mutant Kras in murine initiation models. As KRAS^G12R^ is unable to activate PI3K signaling (11), these data provide a clear rationale for the limited ability of Kras^G12R^ to induce tumorigenesis in murine GEMMs.

### KRAS^G12R^ promotes decreased levels of KRAS hallmark activation relative to KRAS^G12D^

As our data suggest that human PDAC does not require KRAS-mediated activation of PI3K signaling, it is unclear why KRAS^G12R^-mutant patients have been reported to have less aggressive disease and increased overall survival (17). Previously, *KRAS*^*G12R*^ patients were reported to harbor an increased frequency of comutations in tumor suppressor genes or genes within the PI3K pathway in comparison with *KRAS*^*G12D/V*^ (17, 32). We examined the AACR Project GENIE data registry (6,462 samples of patients with PDAC) and found no significant differences in tumor suppressor or PI3K pathway comutations (Supplementary Fig. S5A and S5B).

**Figure 5.**
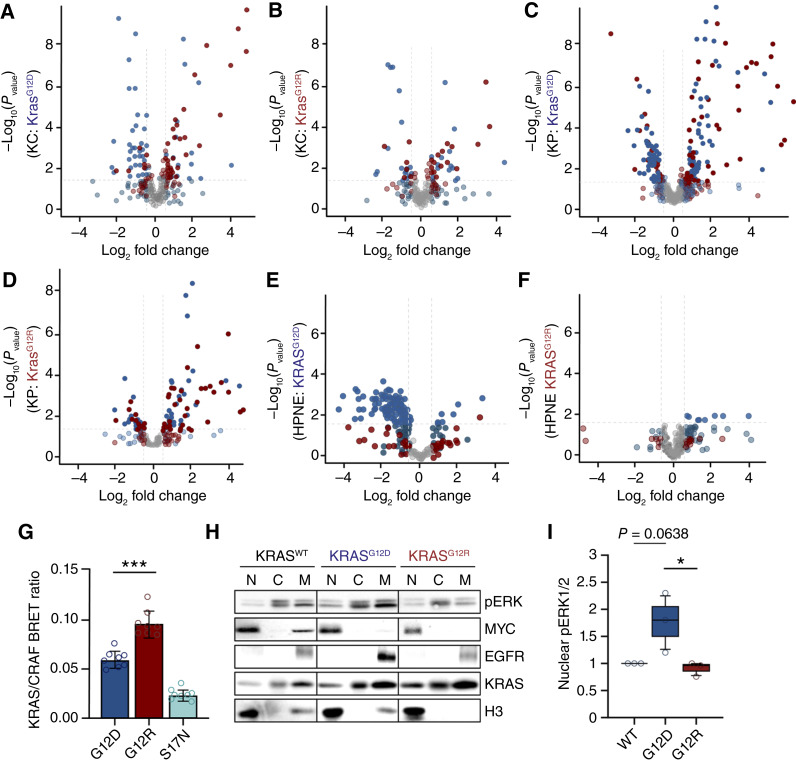
The PDAC KRAS transcriptional signature is weakly activated in KRAS^G12R^-mutant model systems. **A,** Volcano plot with changes in the 200 KRAS-dependent upregulated (red) and downregulated (blue) genes in the PKS in Kras^G12D/+^;Pdx1-Cre 4-week-old murine pancreas compared with Kras^WT^;Pdx1-Cre. All nonsignificant changes are denoted in gray (*P*_adj._ > 0.05), and lighter colors indicate nonsignificant <0.5 log_2_ fold change. The dotted lines represent the cutoff of 0.05 adjusted *P* value and ±0.5 log_2_ fold change. **B,** Volcano plot with PKS changes in Kras^LSL-G12R/+^;Pdx1-Cre 4-week-old murine pancreas compared with Kras^WT^;Pdx1-Cre control pancreas using methods outlined in **A**. **C,** Volcano plot with changes in PKS in ductal pancreatic organoids Kras^LSL-G12D/+^:Cas9-P2A-Cre & Trp53 sgRNA (KP-G12D) compared with KP-WT using methods outlined in **A**. **D,** Volcano plot with changes in PKS in KP-G12R ductal pancreatic organoids compared with KP-WT using methods outlined in **A**. **E,** Volcano plot showing PKS changes in the KRAS^G12D^-expressing hTERT-HPNE E6/E7 cell line in comparison with the HPNE-EV control using methods outlined in **A**. **F,** Volcano plot showing PKS changes in the KRAS^G12R^-expressing hTERT-HPNE E6/E7 cell line in comparison with the HPNE-EV control using methods outlined in **A**. **G,** Quantification of BRET measurements between CRAF and KRAS^G12D^, KRAS^G12R^, or KRAS^S17N^. BRET signal serves as a proxy for binding affinity. Data represent the average of three independent experiments, each with eight technical replicates. **H,** Representative immunoblot of HA-KRAS^WT/G12D/R^-expressing HPNE cells that were fractionated using a subcellular protein fractionation kit. Normalized portions of each extract were loaded for Western blot analysis (*n* = 3). C, cytoplasmic fraction; M, membrane fraction; N, nuclear fraction. **I,** Box-and-whisker plot quantifying nuclear pERK1/2 levels from HPNE subcellular fractionation. pERK1/2 was normalized to the loading control in the nuclear fraction. Murine RNA data were extracted from the Sequence Read Archive under accession PRJNA578549. GSEA was used to compare PKS between KRAS^G12D^ and KRAS^G12R^. All comparisons were significant (*P* < 0.01) except for HPNE PKS UP. *P* values were calculated using one-way ANOVA. *, *P* < 0.05; ***, *P* < 0.0001. Error bars, mean ± SEM. Statistical tests were performed using R version 4.2.2.

Recently, we established a PDAC *KRAS*-dependent transcriptomic gene signature (PKS; refs. 33, 34). We applied this gene signature to assess KRAS^G12R^ versus KRAS^G12D^ signaling outputs in several pancreatic models to determine whether KRAS-RAF-ERK signaling was similar between the mutants (Supplementary Fig. S5C). We utilized the top 200 KRAS-dependent genes upregulated in PKS and labeled them as PDAC “UP” (red), along with the 200 most significantly downregulated KRAS-dependent genes labeled as PDAC “DN” (blue). On the volcano plot, red dots on the right represent genes upregulated by KRAS in the murine and human models, whereas blue dots on the left represent genes downregulated by KRAS in these systems. This analysis enables direct comparison of KRAS-driven gene expression changes across different mutations and cell models. First, we utilized a publicly available RNA-seq dataset derived from *Kras*^*G12D*^- and *Kras*^*G12R*^-mutant mouse pancreases. Our PKS analysis showed robust activation of Kras-driven transcription in *Kras*^*G12D*^ murine pancreatic models (Fig. 5A), whereas the PKS in *Kras*^*G12R*^-mutant murine pancreas was significantly reduced (Fig. 5B).

We then analyzed an additional RNA-seq dataset derived from pancreatic ductal organoids from *Kras*^*LSL-G12D/R*^ mice (18). In these organoids, *Trp53* was deleted by CRISPR/Cas9 gene editing (KP-Mut). This model system also showed robust PKS activation in KP-G12D (Fig. 5C). Again, KP-G12R organoids failed to promote a similar PKS (Fig. 5D).

Next, we tested a human model to compare *KRAS*^*G12D*^ and *KRAS*^*G12R*^ in transduced HPNE cell lines (35, 36). We titrated ectopic KRAS to endogenous levels using a doxycycline-inducible *KRAS* expression vector (Supplementary Fig. S5D). We then measured transcriptional changes in the PKS induced by short-term expression of KRAS^G12D^ and KRAS^G12R^ compared with the empty vector control (Supplementary Fig. S5E). We detected a robust downregulation of the PKS in HPNE-KRAS^G12D^ but not in HPNE-KRAS^G12R^ (Fig. 5E and F). Taken together, these analyses indicate that *KRAS*^*G12R*^ is significantly reduced in its ability to drive the PKS in pancreatic cells of both mouse and human origin.

### KRAS^G12R^ has significantly higher binding to CRAF than KRAS^G12D^

The PKS transcriptional profile is largely driven by KRAS activation of the RAF-MEK-ERK pathway (33, 34). We investigated the binding of KRAS^G12D^ and KRAS^G12R^ to CRAF using the nano-luciferase-BRET assay (37). Surprisingly, we observed that KRAS^G12R^ demonstrated a significantly higher affinity for CRAF than KRAS^G12D^ (Fig. 5G; Supplementary Fig. S5F). As ERK can only elicit transcriptional changes when phosphorylated and in the nucleus (38), we examined the nuclear fraction from HPNE cells and observed significantly lower levels of nuclear phosphorylated ERK1/2 in HPNE-KRAS^G12R^ (Fig. 5H and I). Thus, KRAS^G12R^ seems capable of activating the ERK/MAPK pathway but fails to drive ERK1/2 transcriptional changes to a level similar to that of KRAS^G12D^.

### KRAS^G12R^ PDAC has distinct alterations to the tumor microenvironment–related transcriptomic signature

**Figure 6.**
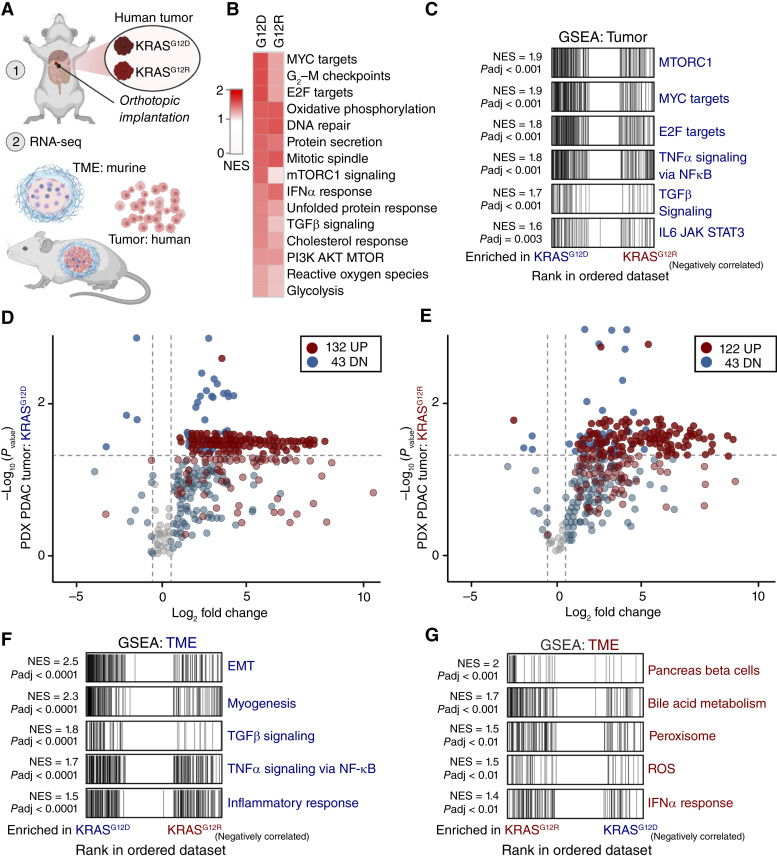
*KRAS* ^*G12R*^-mutant PDAC exhibits a distinct TME. **A,** Schematic of the PDX model. **B,** Heatmap of NESs from GSEA hallmark analysis using the Molecular Signatures Database (MSigDB) of PDX KRAS^G12D/R^, compared with transcriptomics from three healthy pancreas samples. **C,** Significant MSigDB hallmark pathways from GSEA comparing PDX KRAS^G12D^ tumors with KRAS^G12R^ tumors. **D,** Volcano plot showing PKS changes in KRAS^G12D^ PDX PDAC compared with normal pancreas using methods outlined in Fig. 4A. **E,** Volcano plot of RNA from KRAS^G12R^ PDX PDAC tumors compared with normal pancreas, as outlined in Fig. 4A. **F,** Significant hallmark pathways in the TME of PDX-PDAC KRAS^G12D^ compared with PDX PDAC KRAS^G12R^ using GSEA hallmark analysis. NES and adjusted *P* values are listed for all corresponding pathways. **G,** Significant hallmark pathways in the TME of PDX PDAC KRAS^G12R^ compared with PDX PDAC KRAS^G12D^ using GSEA hallmark analysis. NES and adjusted *P* values are listed for all corresponding pathways. ROS, reactive oxygen species.

We next used transcriptomic analysis from the inducible HPNE model to determine whether KRAS^G12R^ and KRAS^G12D^ model systems have a KRAS mutant–dependent signature. HPNE KRAS^G12D^ and KRAS^G12R^ cells separately cluster on a PCA plot (Supplementary Fig. S6A). Further, unbiased clustering demonstrated that KRAS^G12R^ significantly upregulated signatures in clusters 1 and 4, which are associated with increased signaling receptor activity, immune system processes, cytokine/chemokine activity, and inflammatory response (Supplementary Fig. S6B–S6D). We then wanted to compare the transcriptional signature of resected human *KRAS*^*G12R*^ tumors against *KRAS*^*G12D*^ tumors. Direct examination of RNA-seq data in PDAC presents challenges because of the high stromal content and low tumor volume. To address this, we orthotopically implanted human tumors (PDX) into immunocompromised mice, as previously published (21, 24). Utilizing PDXs for both *KRAS*^*G12D*^- and *KRAS*^*G12R*^-mutant tumors, we analyzed the transcriptome from the human tumor cells and the murine tumor microenvironment (TME; Fig. 6A).

To focus on the tumor-intrinsic differences, we first compared the gene expression profiles of *KRAS*^*G12D*^ and *KRAS*^*G12R*^ human tumors with those of normal pancreas using GSEA. The top 15 significantly upregulated pathways were similar; however, the NESs of human *KRAS*^*G12D*^ PDX tumors were moderately higher than those of *KRAS*^*G12R*^ when compared with normal pancreas (Fig. 6B). Enrichment of the PI3K/AKT/mTOR pathway was similar in both KRAS^G12D^ and KRAS^G12R^ human PDX models (Fig. 6B), which is not what was previously observed in murine organoids (18). As KRAS^G12R^ does not activate PI3K and there are no differences in PI3K pathway activation between KRAS mutants, we conclude that KRAS is not a major driver of the PI3K pathway in established human PDAC.

We then directly compared the *KRAS*^*G12D*^ with *KRAS*^*G12R*^ PDX tumors. The *KRAS*^*G12D*^ tumors exhibited significant enrichment in mTORC1 signaling, MYC targets, and E2F targets (Fig. 6C). *KRAS*^*G12R*^ tumors did not show any significant enrichments when compared with *KRAS*^*G12D*^. We then performed the PKS with KRAS^G12D^ and KRAS^G12R^. Both KRAS-mutant tumors showed upregulation of the PKS pathway (Fig. 6D and E), in contrast to our earlier time point HPNE model (Fig. 5E and F). Interestingly, both *KRAS*^*G12D*^ and *KRAS*^*G12R*^ led to few significantly downregulated genes in the PKS pathway in the PDX models. Thus, we concluded that the differences between the PKS are more pronounced at earlier time points between *KRAS*^*G12D*^ and *KRAS*^*G12R*^ in human PDAC.

To examine *KRAS* mutant–specific extrinsic differences, we performed RNA-seq analysis to probe the murine TME from *KRAS*^*G12D*^ and *KRAS*^*G12R*^ PDX tumors. The TME from *KRAS*^*G12D*^ tumors was significantly enriched for genes related to epithelial–mesenchymal transition (EMT), myogenesis, TGFβ signaling, and inflammatory signaling (Fig. 6F). In contrast, the TME from *KRAS*^*G12R*^ PDX tumors showed significant differences compared with *KRAS*^*G12D*^ in numerous pathways, including reactive oxygen species, IFNα response, and pancreatic beta cells (Fig. 6G).

In summary, KRAS^G12D^ and KRAS^G12R^ did not drive higher levels of PI3K/AKT/MTOR enrichment pathways in human HPNE or PDX models (Supplementary Fig. S6E and S6F). Combined with our prior cell model analyses, these data suggest that the predominant tumor cell–intrinsic mechanism to explain the reduced oncogenesis of the KRAS^G12R^ allele stems from its inability to drive ERK/MAPK transcriptional changes.

### KRAS^G12R^-mutant human PDAC has reduced migration and collagen deposition

**Figure 7.**
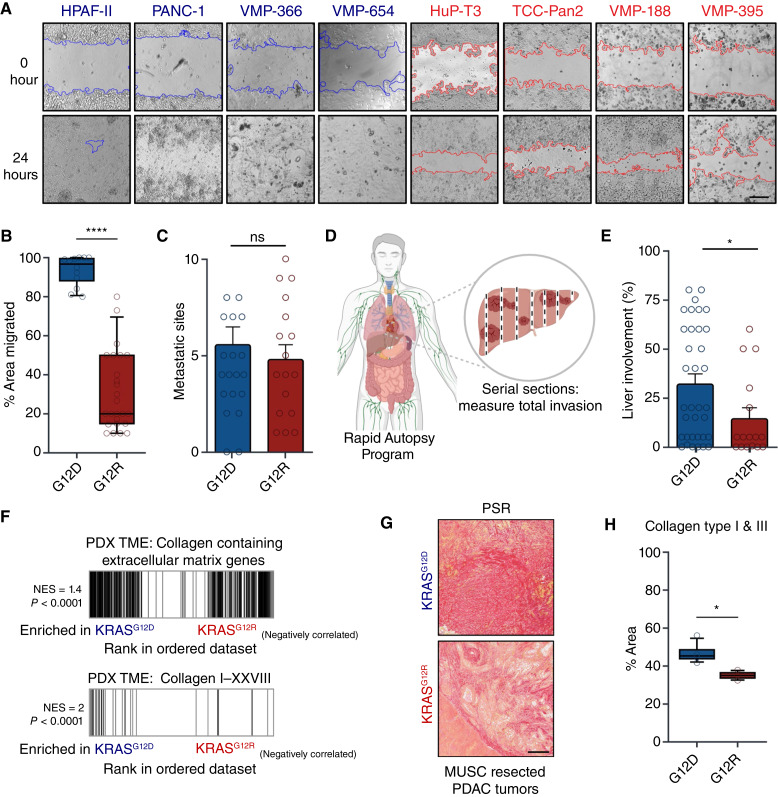
*KRAS* ^*G12R*^-mutant cell lines exhibit reduced migration and collagen deposition. **A,** Representative images of migration assays from a panel of *KRAS*^*G12D*^ and *KRAS*^*G12R*^ PDAC cell lines. Scale bar, 50 μm. **B,** Box-and-whisker plot showing quantification of migration assays from **A**. Mean values are plotted, with each data point representing the percentage of area migrated from at least three technical replicates from three separate experiments for each cell line. **C,** Number of metastatic sites in each patient across indicated KRAS mutations. **D,** Schematic of the rapid autopsy program and how total liver involvement is examined for invasion. **E,** Liver involvement of metastatic lesions. For **C** and **E**, mean values are plotted, with each data point represented by a circle. *KRAS*^*G12D*^, *n* = 37; *KRAS*^*G12R*^, *n* = 17. **F,** Significant Molecular Signatures Database Gene Ontology pathway collagen containing extracellular matrix genes and all collagen genes from GSEA comparing PDX *KRAS*^*G12D*^ tumors with *KRAS*^*G12R*^ tumors. NES and *P* values are listed by corresponding pathways. **G,** Representative brightfield and polarized light images of collagen structures of *KRAS*^*G12D/R*^ using PSR. Polarized light images are representative and at 20× magnification. Scale bar, 300 μm. **H,** Box-and-whisker plot showing quantification of PSR in *KRAS*^*G12D/R*^ resected tumor samples (*n* = 3). Mean values are plotted, with each data point representing the percentage of the area of collagen normalized to the total tumor size. All *P* values were calculated using the Student *t* test comparing G12D and G12R. *, *P* < 0.05; ****, *P* < 0.00001; ns, nonsignificant, *P* > 0.05. Error bars, mean ± SEM.

Our observation that *KRAS*^*G12R*^ PDX models have reduced EMT pathway signatures aligns with recent findings from both bulk transcriptomic analyses of *KRAS*^*G12R*^ patients and an orthotopic murine model, both of which demonstrated decreased migration in *KRAS*^*G12R*^ (17). We validated these findings in CCLs by examining the migratory capacity in a panel of *KRAS*^*G12D*^ and *KRAS*^*G12R*^ pancreatic cancer cells. We observed significantly reduced migration in the *KRAS*^*G12R*^ CCLs (Fig. 7A and B; Supplementary Fig. S7A). In agreement, the *KRAS*^*G12R*^ “RASless” MEFs showed a significantly decreased migration capacity in comparison with *KRAS*^*G12D*^, whereas the faster-proliferating *BRAF*^*V600E*^ MEFs exhibited migration rates similar to *KRAS*^*G12R*^ (Supplementary Fig. S7B and S7C).

We then asked whether metastasis differed between *KRAS*^*G12D*^- and *KRAS*^*G12R*^-mutant tumors in patients with late-stage PDAC. Using samples from the University of Nebraska Rapid Autopsy Program, we compared the number of metastatic sites across more than 12 tissues. *KRAS*^*G12R*^ patients had no significant differences in the number of metastatic sites at mortality (Fig. 7C). The liver is the most common metastatic site in PDAC; therefore, we obtained serial sections from autopsy patients and used them to estimate the percentage of metastatic involvement. *KRAS*^*G12R*^ patients had a significant decrease in metastatic involvement in the liver (Fig. 7D and E). The underlying mechanisms remain unclear, as this could reflect an impaired ability of tumor cells to disseminate and colonize the liver or be due to differences in the metastatic niche, which were not directly examined.

As the PDAC TME is rich in collagen, we examined collagen mRNA levels in the orthotopic PDX models, assessing contributions from both the tumor compartment and the surrounding TME separately. *KRAS*^*G12D*^ and *KRAS*^*G12R*^ tumor cells showed no significant differences in collagen gene expression (Supplementary Fig. S7D); however, the TME displayed a significant enrichment of collagen mRNA in *KRAS*^*G12D*^ samples relative to the *KRAS*^*G12R*^ samples (Fig. 7F). Utilizing human patient biopsies from Hollings Cancer Center, we quantified a cohort of three *KRAS*^*G12D*^ and three *KRAS*^*G12R*^ resected tumors for types I and III collagen, the predominant fibrillar collagens in PDAC. We observed a significant decrease in PSR staining, which specifically labels collagen I and III, in the *KRAS*^*G12R*^ patients (Fig. 7G and H). We also observed an increase in crimped collagen structures in *KRAS*^*G12R*^ tumors, suggesting that collagen organization may differ between *KRAS*^*G12R*^ and *KRAS*^*G12D*^ tumors (Supplementary Fig. S7E). This observation highlights the need to obtain quantitative measurements of collagen composition and structure in future studies. Overall, we conclude that *KRAS*^*G12R*^ human tumors promote lower levels of collagen deposition in the TME.

## Discussion

*KRAS* ^*G12R*^ is the third most frequent KRAS mutation in PDAC yet is rare in other cancer types. Patients with PDAC harboring the *KRAS*^*G12R*^ mutation have a better response to chemotherapy and longer overall survival (17, 32, 39). We evaluated *KRAS*^*G12R*^ in a series of GEMMs, murine and human cell lines, and human PDAC samples to define the mechanisms that make KRAS^G12R^ such a unique KRAS-mutant protein. We demonstrated that murine models are more reliant on Kras for PI3K/AKT activation, whereas human PDAC has a reduced requirement for KRAS to activate the PI3K/AKT pathway. Further, we found that KRAS^G12R^, despite a clear ability to activate cytoplasmic ERK/MAPK signaling, had reduced nuclear ERK translocation, resulting in impaired transcriptional activation of ERK targets in both murine and human PDAC systems. Finally, our work demonstrates that *KRAS*^*G12R*^ promotes a distinct collagen deposition pattern in human PDAC samples and differences between mutations persist at stage IV. Together, our findings reveal critical new differences between murine models of PDAC initiation and tumor maintenance in PDAC and define new features that earn KRAS^G12R^ its “atypical” moniker.

KRAS GEMMs have provided key insights into tumorigenesis and the treatment of PDAC (40), and numerous models have been developed by adding comutations, including Trp53 (26), CDKN2A (41), and PTEN (42). Many of these models harbored *Kras*^*G12D*^. Recently, *Kras*^*G12C*^, *Kras*^*G12R*^, and *Kras*^*G13D*^ were introduced in pancreatic and colon GEMMs (18). Although *Kras*^*G12C*^ and *Kras*^*G13D*^ were able to promote PanIN formation in this context, the *Kras*^*G12R*^ mutation remained stubbornly recalcitrant to disease initiation in the absence of the LSL-mKate2 transgenic reporter allele (18). Herein, we report that the K^R^P48 model developed ubiquitous vacuole formation and hyperplasia in the pancreas and that tumors developed in 10% of K^R^P48 mice at 1 year of age. This study represents the first reported instance of *de novo* tumor formation in a true *Kras*^*G12R*^ GEMM.

As different tissues have different susceptibilities to KRAS-driven tumorigenesis (19), we generated a Kras^G12R/+^;*Rosa26*^*CRE-ERT2/+*^ mouse model to better define the limitations of the *Kras*^*G12R*^ allele. The *Kras*^*G12R/+*^*;Rosa26*^*CRE-ERT2*^ model was resistant to tumor formation in all tissues, with all mice surviving over 1 year of age and no outward appearance of disease, in stark contrast to the *Kras*^*G12D*^ counterpart. Previously, the Rosa26 murine model was susceptible to multifocal disease even when using the strongly activating *Kras*^*Q61R*^ allele (19), suggesting that the inability of *Kras*^*G12R*^ to drive disease is due to poor oncogenic capacity. Combined, these GEMMs agree with a previous report that defined the *KRAS*^*G12R*^ allele as a “weak” oncogene (17), and we identified two distinct mechanisms that limit the tumorigenic potential of the *Kras*^*G12R*^ allele in murine models.

One mechanism for the “weakness” of the *Kras*^*G12R*^ allele in murine models of tumor initiation is the apparent requirement for Kras-mediated activation of the PI3K pathway. However, utilizing *Kras*^*G12R*^-mutant murine pancreatic organoids and orthotopic implantation can overcome this initiation defect (17, 18). Whether the initiation defect is overcome via the use of growth factors to stimulate the PI3K pathway or due to ductal differentiation of the organoids remains unknown. It is possible that ductal cells have distinct requirements for PI3K/AKT activation; therefore, crossing *Kras*^*G12R*^ with *Sox9* ductal-specific Cre (43) may provide insight into Kras^G12R^ signaling in the murine context. However, the PI3K pathway is activated independently of KRAS in established human PDAC, allowing KRAS^G12R^ to overcome this weakness at later stages of the disease. Thus, an important conclusion from this study is that the orthotopic model may more accurately reflect human PDAC as KRAS^G12R^ is capable of driving tumorigenesis and recapitulates a “weaker” phenotype in the orthotopic setting (17). Additionally, the strong reliance on Kras for AKT activation for tumor initiation in the KPC model systems highlights a striking difference between models of initiation and established disease, suggesting that KRAS does not directly mediate the majority of AKT activation in established human PDAC. This is supported by a separate study, wherein we demonstrate that human PDAC cells have oxidized and thereby inactivated PTEN, which results in intrinsically elevated PI3K activity. Furthermore, nutrient deprivation (specifically glucose and glutamine) augments PTEN oxidation, and sustained nutrient deprivation lowers overall cellular PTEN levels, thereby promoting increased resistance to PI3K inhibition (44).

A second mechanism for the weakness of the *Kras*^*G12R*^ allele in both murine PDAC and human PDAC is the reduced ability of *KRAS*^*G12R*^ to promote downstream ERK/MAPK signaling. Despite measuring tight binding to RAF1 and strong ERK/MAPK phosphorylation, *KRAS*^*G12R*^ failed to promote activated nuclear ERK. Recently, we reported a detailed analysis of the effect of mutant KRAS signaling and transcriptional regulation, termed the PKS (33, 34). In agreement with the reduced nuclear ERK activity, we determined that *KRAS*^*G12R*^ had a reduced capacity to promote the PKS in model cell systems. Our early time point HPNE model system had much larger differences in PKS across KRAS^G12D^ and KRAS^G12R^. However, in our orthotopic PDXs derived from resectable human tumors, the *KRAS*^*G12R*^ and *KRAS*^*G12D*^ tumor transcriptional networks converged to have more similarities, suggesting that human tumors overcome the limitations intrinsic to *KRAS*^*G12R*^. If PI3K activation is required for human PDAC initiation, it remains a priority in the field to understand how KRAS^G12R^ overcomes this limitation.

Further, our migration analysis in human CCLs supports the findings from a recent clinical study suggesting that *KRAS*^*G12R*^ tumors are less likely to promote distal metastasis (17). The reduced collagen deposition in *KRAS*^*G12R*^ human tumors suggests that KRAS mutant–specific signaling differences persist at resection. Although the role of collagen in PDAC is debated, previous reports have shown that collagen can promote gemcitabine resistance, worse prognosis, and increased metastasis and is a potential source of metabolic fuel for cancer cells (45). Thus, these observations suggest that the improved therapeutic response and increased survival of *KRAS*^*G12R*^-mutant patients may be due to the decreased collagen deposition induced by the KRAS^G12R^ mutant protein. A crucial future direction will be to investigate the collagen-related processes and determine whether collagen deposition in the TME is differentially driven by KRAS mutants, as our study suggests.

Finally, our results from the rapid autopsy program revealed that *KRAS*^*G12R*^ maintains the capacity to metastasize at a similar frequency as KRAS^G12D^ although the metastatic niche in the liver is significantly different. Combined, our data suggest that mutant-selective signaling differences driven by KRAS^G12D^ and KRAS^G12R^ decrease as the tumors progress, suggesting that mutation-selective therapies are only likely to be effective at early stages.

As exciting as the *de novo* model for Kras^G12R^-mediated PDAC tumorigenesis may be, this GEMM is limited by its low tumor penetrance and delayed onset, as well as potential confounding factors, such as advanced age and comorbidities from Trp53 hemizygosity, making this model challenging for use in the field. Other studies have demonstrated an increase in pancreatic lesions in a Kras^G12R^ GEMM upon constitutive Akt activation (via the myristoylated Akt transgene; bioRxiv 2022.06.24.497515), and we have separately demonstrated that PI3K signaling is KRAS independent in human PDAC due in part to the inactivation of PTEN by intramolecular disulfide bond formation and elevated PI3K isoform expression (44), a feature that is not observed in the murine system, further alluding to limitations about Akt signaling in the mouse.

Together, these data reinforce the idea that *KRAS* mutations drive distinct processes that can be used as biomarkers to inform clinical decisions, and *KRAS*^*G12R*^ patients may be more likely to respond or less likely to metastasize further if treated with neoadjuvant chemotherapy. However, more research is required to fully dissect the therapeutic window in which *KRAS*^*G12R*^ patients would be the most vulnerable.

## Supplementary Material

Supplemental Table S1 Animals used for KRAS mouse models

Supplemental Table S2 Key Resources

Supplemental Figure S1 Engineering a KrasLSL-G12R/+ murine model.

Supplemental Figure S2 KrasLSL-G12D/+;Trp53LSL-R172H/+;p48LSL-Cre-ERTM promotes PDAC and liver metastases whereas KrasLSL-G12R/+;Trp53LSL-R172H/+;p48LSL-Cre-ERTM drives inflammation.

Supplemental Figure S3 Full body KrasLSL-G12D;Rosa26Cre-ER/LSL-EYFP increases endocrine pancreatic vacuoles and drives changes in the lung and spleen.

Supplemental Figure S4 KRASG12D–mutant cell lines have increased proliferation rates compared to KRASG12R.

Supplemental Figure S5 The frequency of co-mutations in tumor suppressor genes is not dependent on KRAS mutation status in human pancreatic cancer patients in the AACR Project GENIE dataset.

Supplemental Figure S6 KRAS mutations promote differential transcriptional outcomes.

Supplemental Figure S7 KRASG12R-mutant cell lines have reduced migration.

## Data Availability

The RNA-seq data analyzed in this study are publicly available in the Gene Expression Omnibus at GSE252909 and the Sequence Read Archive at PRJNA578549. All other raw data generated in this study are available from the corresponding author upon request.
