# Supplementary material for: KRASG12R-Mutant Pancreatic Cancer Features Limited ERK/MAPK Transcriptional Activity and a Distinctive Tumor Microenvironment
Source: Cancer Res. 2026 Jan 13;86(8):1868–82. doi: 10.1158/0008-5472.CAN-25-2630 (PMC13080325; doi:10.1158/0008-5472.CAN-25-2630)
Supplement: Supplemental Table S1 — Animals used for KRAS mouse models [file can-25-2630_supplemental_table_s1_suppst1.pdf]

Supplemental Table S1. Numbers and sex of mice used in study

	KP48	KRC
**Control**	16 MALE<br>10 FEMALE	7 MALE<br>8 FEMALE
**KRAS^{G12D}**	Kras$^{LSL\text{-}G12D/+}$;Trp53$^{LSL\text{-}R172H/+}$;p48$^{CRE\text{-}ERTM}$<br>16 MALE<br>10 FEMALE	Kras$^{LSL\text{-}G12D/+}$;<br>*Rosa26$^{Cre\text{-}ER/LSL\text{-}EYFP}$*<br>7 MALE<br>4 FEMALE
**KRAS^{G12R}**	Kras$^{LSL\text{-}G12R/+}$;Trp53$^{LSL\text{-}R172H/+}$;p48$^{CRE\text{-}ERTM}$<br>10 MALE<br>10 FEMALE	Kras$^{LSL\text{-}G12R/+}$;<br>Rosa26$^{Cre\text{-}ERT2}$<br>2 MALE<br>3 FEMALE
