# Supplementary material for: KRASG12R-Mutant Pancreatic Cancer Features Limited ERK/MAPK Transcriptional Activity and a Distinctive Tumor Microenvironment
Source: Cancer Res. 2026 Jan 13;86(8):1868–82. doi: 10.1158/0008-5472.CAN-25-2630 (PMC13080325; doi:10.1158/0008-5472.CAN-25-2630)
Supplement: Supplemental Table S2 — Key Resources [file can-25-2630_supplemental_table_s2_suppst2.pdf]

Supplemental Table S2. Key Resources Table

REAGENT or RESOURCE	SOURCE	IDENTIFIER
**Antibodies**		
KRAS mouse mAb	Sigma Aldrich	WH0003845M1, RRID:AB_1842235
AKT rabbit	Cell Signaling Technology	Cat# 9272, RRID:AB_329827
pAKT(S473) (D9E) XP(R) rabbit mAb	Cell Signaling Technology	Cat# 4060, RRID:AB_2315049
pAKT (T308) rabbit mAb	Cell Signaling Technology	Cat# 2965, RRID:AB_2255933
Vinculin (E1E9V) XP(R) rabbit mAb	Cell Signaling Technology	Cat# 13901, RRID:AB_2728768
P-p44/42 MAPK (T202/Y204) (D13.14.4E) XP(R) rabbit mAb	Cell Signaling Technology	4370, RRID:AB_2315112
P44/42 MAPK (Erk1/2) rabbit Ab	Cell Signaling Technology	Cat# 9102, RRID:AB_330744
MEK 1/2 (D1A5) rabbit mAb	Cell Signaling Technology	Cat# 8727, RRID:AB_10829473
P-MEK 1/2 (S217/221) (41G9) rabbit mAb	Cell Signaling Technology	Cat# 9154, RRID:AB_2138017
GAPDH (14C10) rabbit mAb	Cell Signaling Technology	Cat# 2118, RRID:AB_561053
**Biological samples**		
Pancreatic Ductal Adenocarcinoma resected tumors	This paper	Medical University of South Carolina
**Critical commercial assays**		
Active Ras Detection Kit	Cell Signaling	#8821
NEBNext Poly(A) mRNA Magnetic Isolation Module	New England Biolabs	NEB #E3370
NEBNext Ultra II Directional RNA Library Prep Kit for Illumina	New England Biolabs	E7760S
**RNA Sequencing Data**		
Raw and analyzed RNA data		GEO: GSE252909
Murine RNA-seq		SRA: PRJN578549
**Experimental models: Cell lines**		
293T	ATCC	CRL-3216
VMP-654	UVA	N/A
VMP-366	UVA	N/A
VMP-188	UVA	N/A
VMP-395T	UVA	N/A
AsPC-1	ATCC	CVCL_0152
PANC-1	ATCC	CVCL_0480
TCC-Pan2	Japanese Collection of Research Bioresources Cell Bank	CVCL_3178
KP-2	Japanese Collection of Research Bioresources Cell Bank	CVCL_3004
HuP-T3	ATCC	CVCL_1299
HPAF-II	ATCC	CVCL_0313
SW1990	ATCC	CVCL_1723

hTERT-HPNE E6/E7	ATCC	CVCL_C467
'RASless' mouse embryonic fibrobasts (MEFs)	NIH	
KPC 2838c3		CVCL_YM18
KPC 6419c5		CVCL_YM21
KPC	ATCC	CVCL_1U12
**Experimental models: Organisms/strains**		
B6.129S4-*Kras*^{tm4Tyj}/J	The Jackson Laboratory	RRID:IMSR_JAX:008179 #:008179
B6.129S6(Cg)-*Ptf1a*$^{tm2(cre/ESR1)Cvw}$/J	The Jackson Laboratory	RRID:IMSR_JAX:036577 #:036577
129S-*Trp53*^{tm2Tyj}/J	The Jackson Laboratory	RRID:IMSR_JAX:008652 #:008652
B6.129-*Gt(ROSA)26Sor*$^{tm1(cre/ERT2)Tyj}$/J	The Jackson Laboratory	RRID:IMSR_JAX:008463 #:008463
C57BL/6J	The Jackson Laboratory	RRID:IMSR_JAX:000664
**Oligonucleotides**		
GAAGGCATTTGTGTAGGTCA	Eurofins Genomics	p48 CRE.FOR
GGCCTGAGTGAGGGTTGTGAG	Eurofins Genomics	p48 CRE.REV
TGTCTTTCCCCAGCACAGT	Eurofins Genomics	KRAS.WT.FOR
GCAGGTCGAGGGACCTAATA	Eurofins Genomics	KRAS.MT.FOR
CTGCATAGTACGCTATACCCTGT	Eurofins Genomics	KRASCommon.REV
GTC TGGAATTCCGCAAGCTA	Eurofins Genomics	Y582.Trp53.1
CTTGGAGACATAGCCACACTG	Eurofins Genomics	T035.Trp53.2
AGAGCCAAAGATAAAGGGTAAAAG	Eurofins Genomics	Y583.Trp53.3
CCAGCACAGTGCAGTTTTGA	Eurofins Genomics	G12R_F3
TTACAAGCGCACGCAGACT	Eurofins Genomics	G12R_R3
TCATAGCCTGAAGAACGAGATCAG	Eurofins Genomics	G12R_Primer3
**Recombinant DNA**		
MSCV-Cre-Hygro		Addgene #34565
pCW57.1-Hygro	Created using pCW57.1 for this study	RRID:Addgene #41393
pBABE-neo largeTcDNA		Addgene #1780
**Software and algorithms**		
GraphPad Prism		RRID:SCR_002798
Biorender		RRID:SCR_018361
Vectra Polaris Automated Quantitative Pathology Imaging System		RRID:SCR_025508
ImageJ		RRID:SCR_003070
Partek Genomics Suite		RRID:SCR_011860
Gene Set Enrichment Analysis	Human MSigDB v2024.1	RRID:SCR_003199
Illumina NovaSeq 6000	University of Vanderbilt	RRID:SCR_020150
R Studio	R version 4.4.2	RRID:SCR_001905
Spliced transcript alignment to a reference (STAR)		RRID:SCR_004463
