# Supplementary figures and images for: KRASG12R-Mutant Pancreatic Cancer Features Limited ERK/MAPK Transcriptional Activity and a Distinctive Tumor Microenvironment
Source: Cancer Res. 2026 Jan 13;86(8):1868–82. doi: 10.1158/0008-5472.CAN-25-2630 (PMC13080325; doi:10.1158/0008-5472.CAN-25-2630)

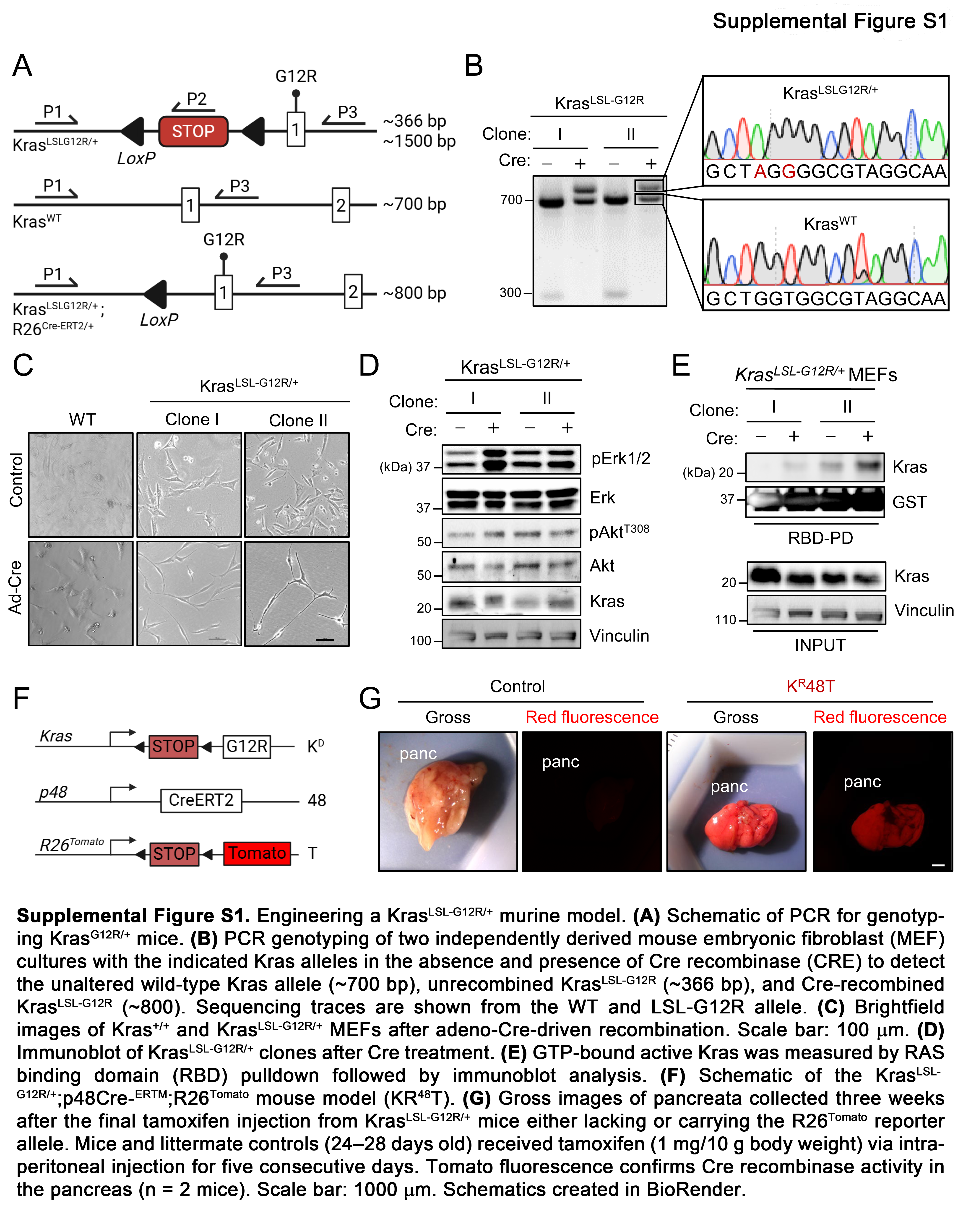

Supplement: Supplemental Figure S1 — Engineering a KrasLSL-G12R/+ murine model. [file can-25-2630_supplemental_figure_s1_suppsf1.png]

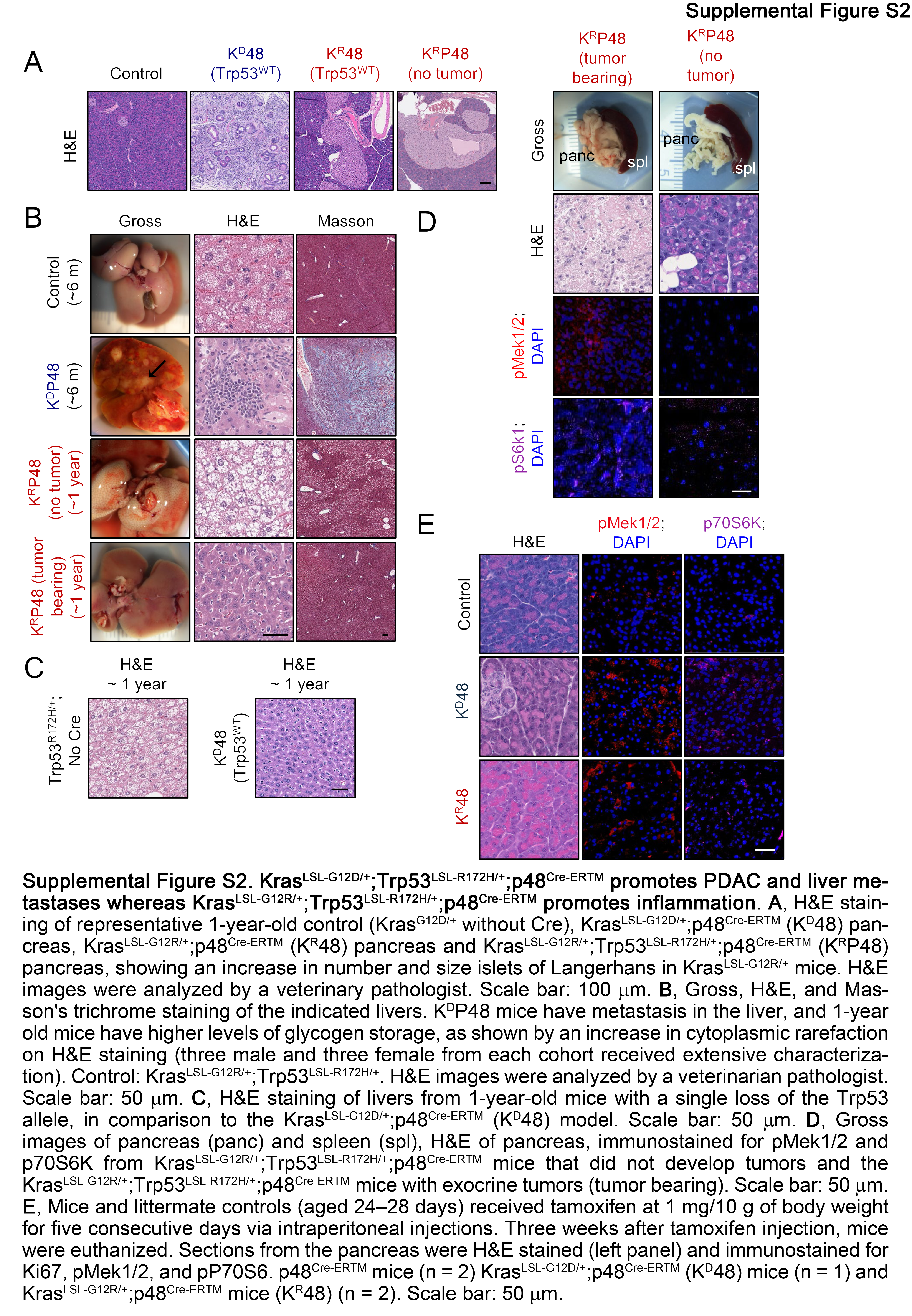

Supplement: Supplemental Figure S2 — KrasLSL-G12D/+;Trp53LSL-R172H/+;p48LSL-Cre-ERTM promotes PDAC and liver metastases whereas KrasLSL-G12R/+;Trp53LSL-R172H/+;p48LSL-Cre-ERTM drives inflammation. [file can-25-2630_supplemental_figure_s2_suppsf2.png]

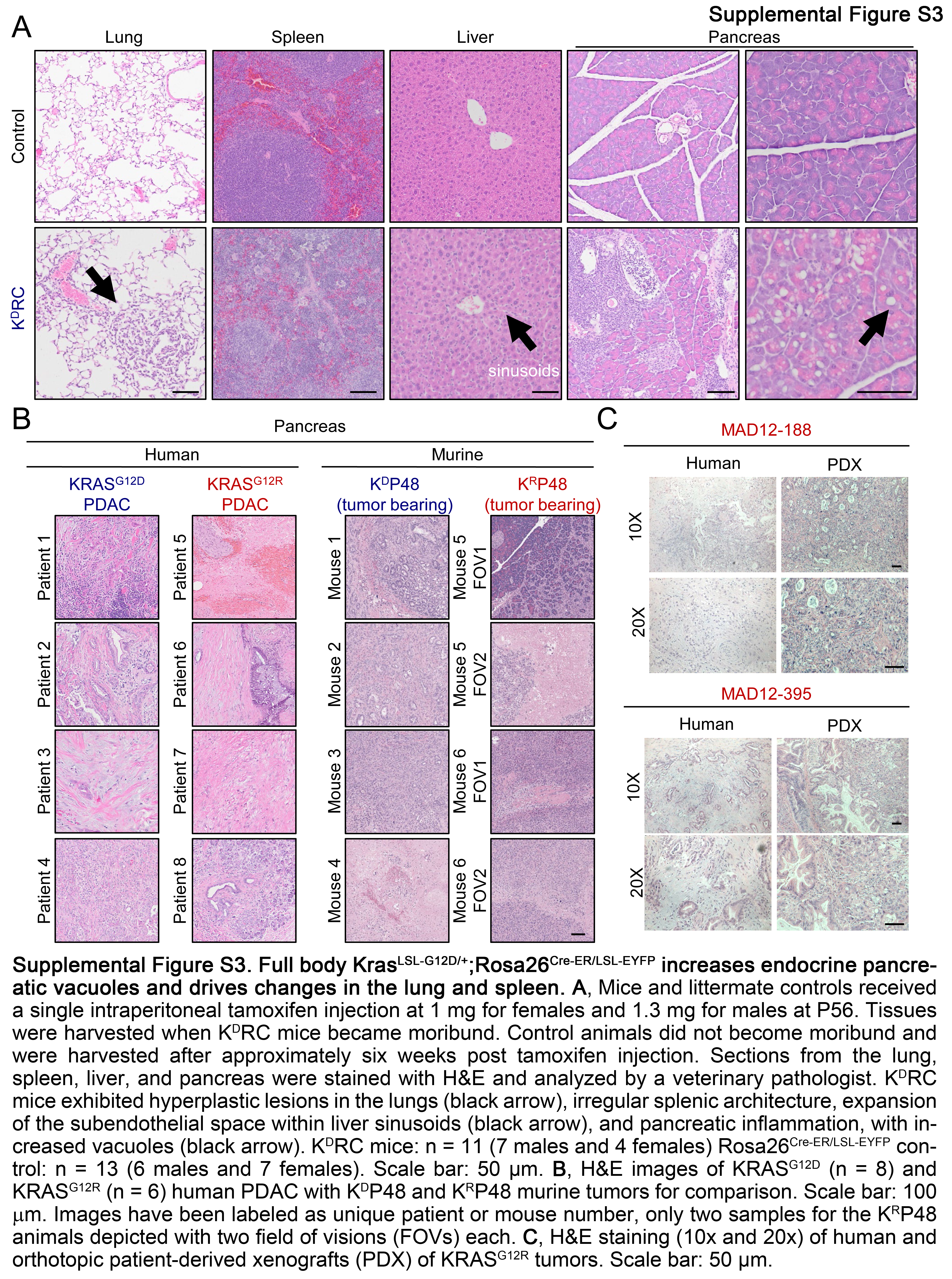

Supplement: Supplemental Figure S3 — Full body KrasLSL-G12D;Rosa26Cre-ER/LSL-EYFP increases endocrine pancreatic vacuoles and drives changes in the lung and spleen. [file can-25-2630_supplemental_figure_s3_suppsf3.png]

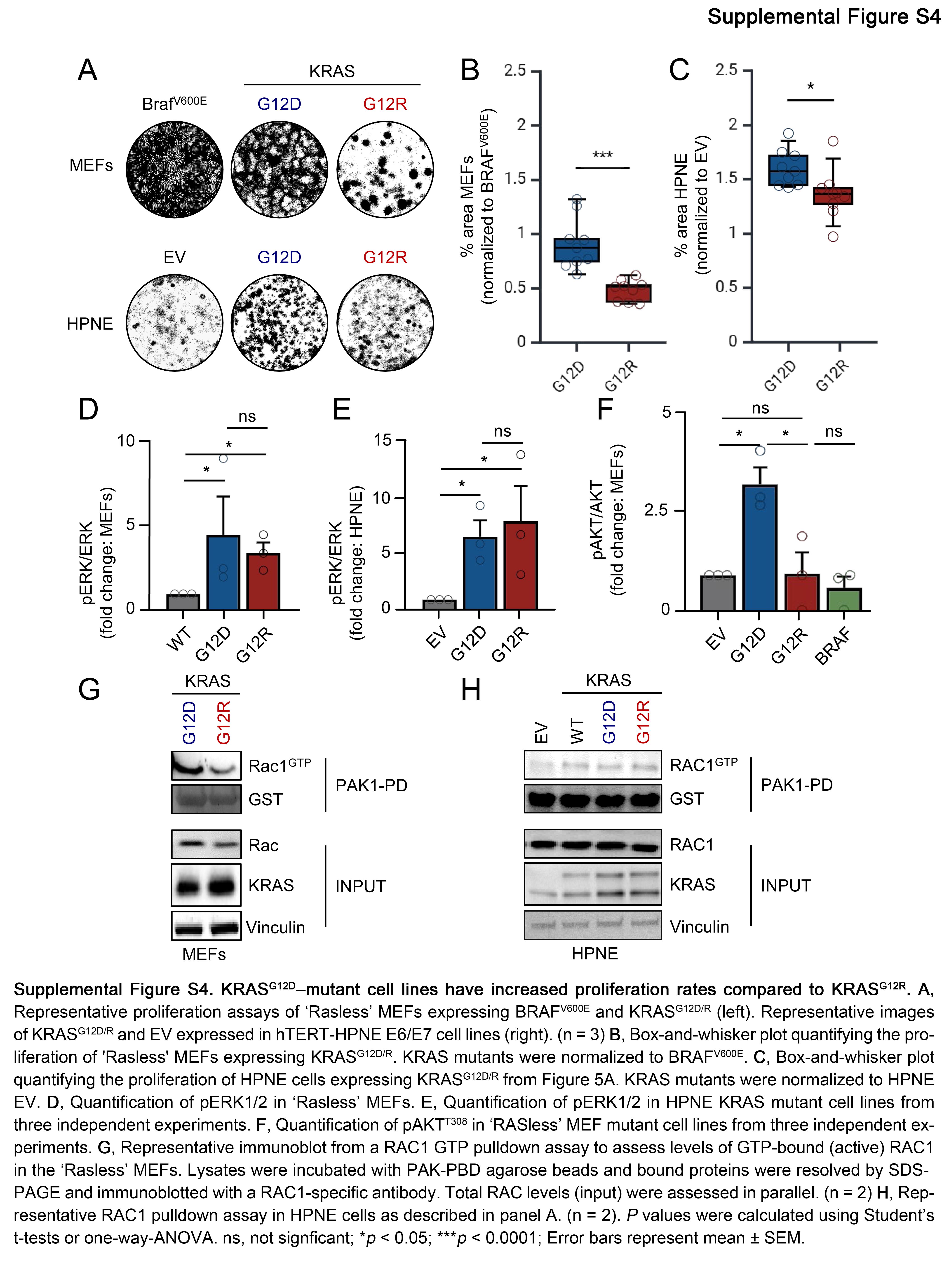

Supplement: Supplemental Figure S4 — KRASG12D–mutant cell lines have increased proliferation rates compared to KRASG12R. [file can-25-2630_supplemental_figure_s4_suppsf4.png]

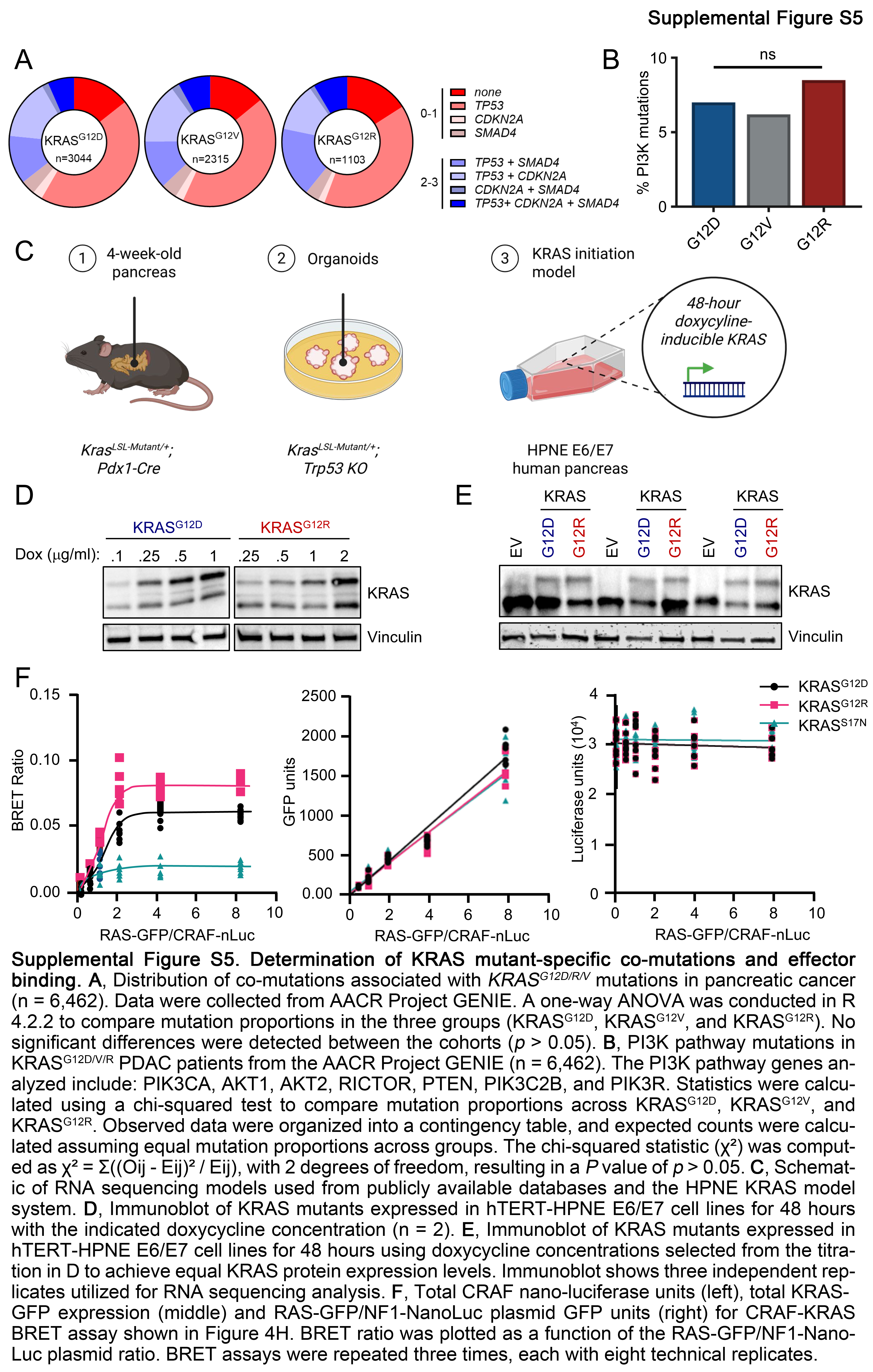

Supplement: Supplemental Figure S5 — The frequency of co-mutations in tumor suppressor genes is not dependent on KRAS mutation status in human pancreatic cancer patients in the AACR Project GENIE dataset. [file can-25-2630_supplemental_figure_s5_suppsf5.png]

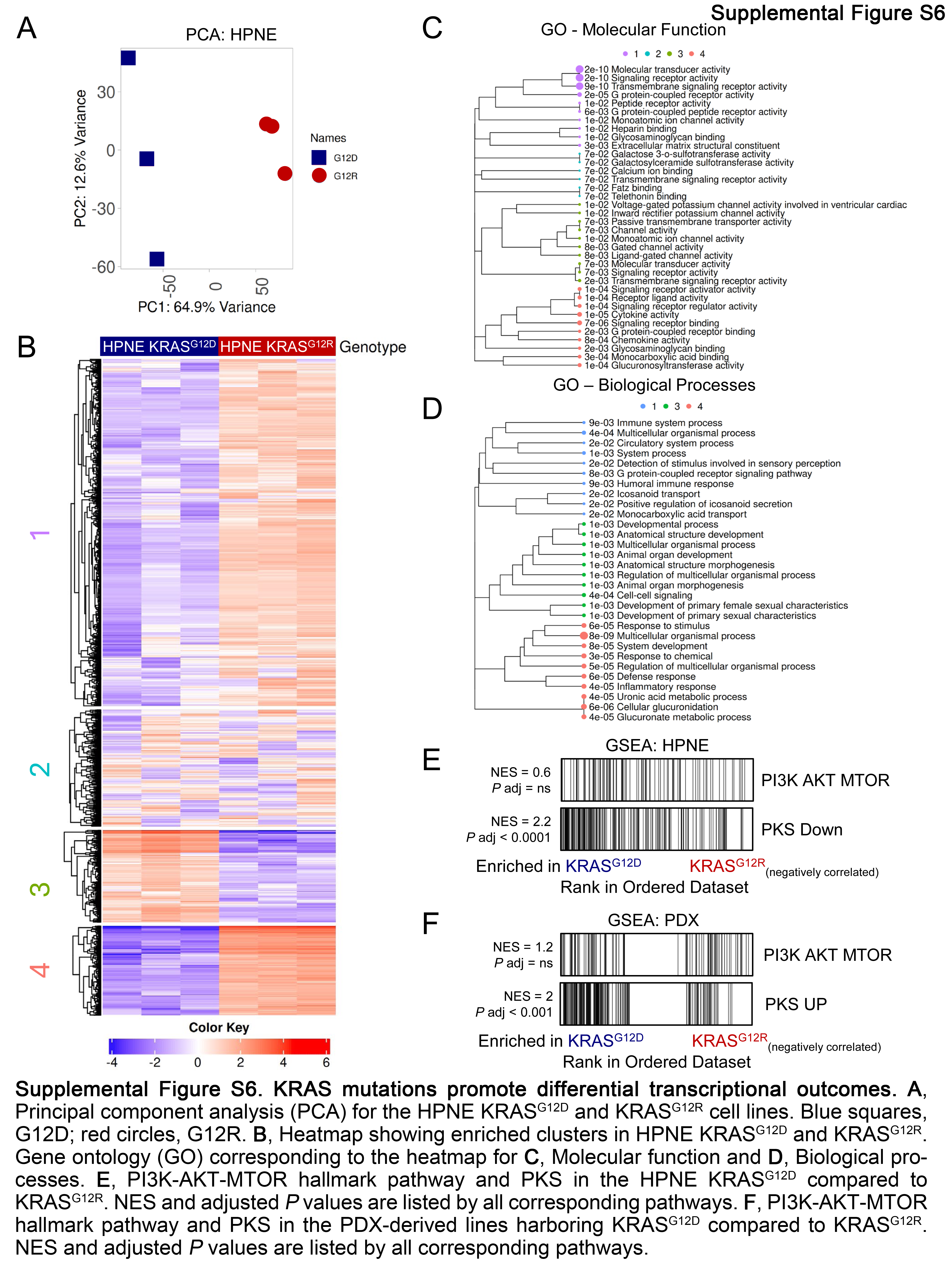

Supplement: Supplemental Figure S6 — KRAS mutations promote differential transcriptional outcomes. [file can-25-2630_supplemental_figure_s6_suppsf6.png]

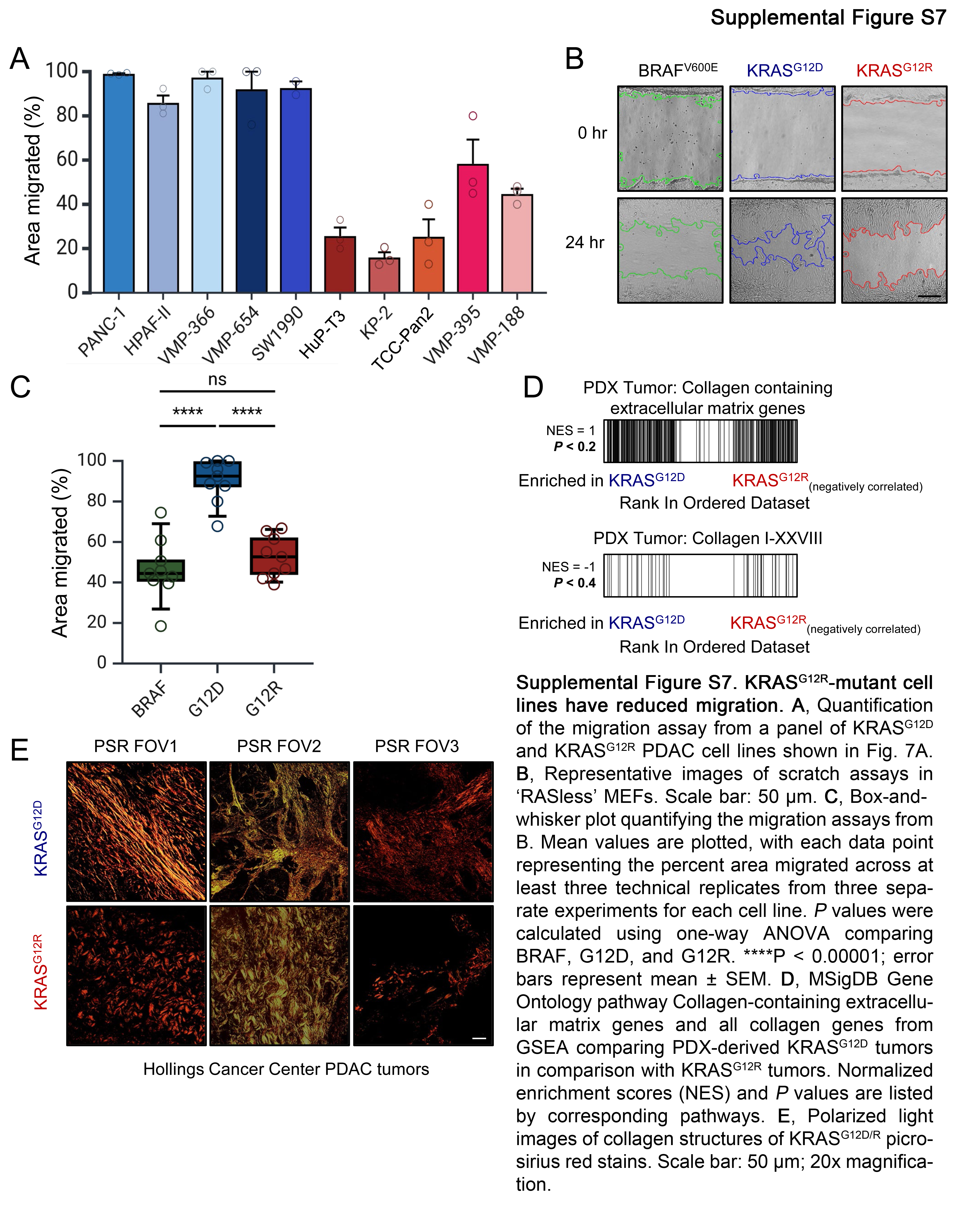

Supplement: Supplemental Figure S7 — KRASG12R-mutant cell lines have reduced migration. [file can-25-2630_supplemental_figure_s7_suppsf7.png]
